# Transcriptional re-programming of insulin B-chain epitope-specific T-follicular helper cells into anti-diabetogenic T-regulatory type-1 cells

**DOI:** 10.3389/fimmu.2023.1177722

**Published:** 2023-04-19

**Authors:** Patricia Solé, Daniel Parras, Jun Yamanouchi, Josep Garnica, Nahir Garabatos, Joel Moro, Javier Montaño, Debajyoti Mondal, César Fandos, Yang Yang, Pau Serra, Pere Santamaria

**Affiliations:** ^1^ Department of Liver, Digestive System and Metabolism, Institut D’Investigacions Biomèdiques August Pi i Sunyer, Barcelona, Spain; ^2^ Department of Microbiology, Immunology and Infectious Diseases, Snyder Institute for Chronic Diseases and Hotchkiss Brain Institute, Cumming School of Medicine, University of Calgary, Calgary, AB, Canada; ^3^ Department of Biochemistry and Molecular Biology, Cumming School of Medicine, University of Calgary, Calgary, AB, Canada

**Keywords:** peptide-major histocompatibility complex (pMHC) molecules, nanomedicine, antigen-specific tolerance, immunoregulation, T-regulatory type 1 (TR1) cells, type 1 diabetes, insulin B-chain

## Abstract

Systemic delivery of nanoparticles (NPs) coated with mono-specific autoimmune disease-relevant peptide-major histocompatibility complex class II (pMHCII) molecules can resolve organ inflammation in various disease models in a disease-specific manner without impairing normal immunity. These compounds invariably trigger the formation and systemic expansion of cognate pMHCII-specific T-regulatory type 1 (TR1) cells. By focusing on type 1 diabetes (T1D)-relevant pMHCII-NP types that display an epitope from the insulin B-chain bound to the same MHCII molecule (IA^g7^) on three different registers, we show that pMHCII-NP-induced TR1 cells invariably co-exist with cognate T-Follicular Helper (TFH)-like cells of quasi-identical clonotypic composition and are oligoclonal, yet transcriptionally homogeneous. Furthermore, these three different TR1 specificities have similar diabetes reversal properties *in vivo* despite being uniquely reactive against the peptide MHCII-binding register displayed on the NPs. Thus, pMHCII-NP treatment using nanomedicines displaying different epitope specificities results in the simultaneous differentiation of multiple antigen-specific TFH-like cell clones into TR1-like cells that inherit the fine antigenic specificity of their precursors while acquiring a defined transcriptional immunoregulatory program.

## Introduction

We have established that systemic injection of nanoparticles (NPs) coated with mono-specific autoimmune disease-relevant peptide-major histocompatibility complex class II (pMHCII) molecules (1) can resolve organ inflammation in various disease models in a disease-specific manner without impairing normal immunity (2, 3). We have documented therapeutic efficacy in spontaneously hyperglycemic nonobese diabetic (NOD) mice, in C57BL/6 mice with experimental autoimmune encephalomyelitis (EAE), in HLA-DR4-transgenic C57BL/10.M mice with experimental arthritis (2), and more recently, NOD.*c3c4*, C57BL/6.Ifng-Δ-ARE^+/–^ and NOD.*Abcb4* ^–/–^ mice with spontaneous primary biliary or sclerosing cholangitis (PBC and PSC, respectively), and NOD and C57BL/6 mice with experimental autoimmune hepatitis (AIH) (3, 4). At the cellular and molecular levels, these compounds trigger prolonged TCR signaling *via* the sustained assembly of TCR microclusters on cognate autoantigen-experienced T-cell precursors (1). These events result in the transcriptional re-programming of these T-cell precursors (2, 5) into interleukin-10 (IL-10)-producing T-regulatory type 1 (TR1) cells, followed by TR1 cell-induced recruitment and re-programming of other cell types of the adaptive and innate immune systems that collectively enforce broad organ-specific immunoregulation (2–4, 6).

Although pMHCII-based nanomedicines trigger the formation and systemic expansion of cognate pMHCII-specific TR1 cells it is unclear whether the cells arising in response to different pMHCII-NP types are transcriptionally similar or different from one another as a function of the pMHCII type used. It also remains to be determined whether the cognate TR1 cell pools arising in response to these compounds do so from a single T-cell clone or from a polyclonal repertoire of epitope-specific T-cell precursors. Furthermore, we do not yet know if these cells exclusively recognize the peptide in the exact same MHCII-binding register as displayed on the pMHCII complexes coated onto NPs, or are inherently cross-reactive against the same or similar epitopes displayed on alternative MHCII-binding registers. Here, we explore these unanswered questions in detail, by focusing on T1D-relevant pMHCII-NP types that display an epitope from insulin bound to the same MHCII molecule (IA^g7^) but on three different registers. Our data show that pMHCII-NP-induced TR1 cells co-exist with cognate T-follicular helper (TFH) cells of quasi-identical clonotypic composition, are transcriptionally homogeneous yet oligoclonal, are specifically reactive against the peptide MHCII-binding register used to elicit them, and have similar therapeutic activity *in vivo*. These data suggest that pMHCII-NP treatment results in the terminal differentiation of multiple antigen-specific TFH cell clones into TR1-like cells with a defined transcriptional program, thus further supporting the translational significance of these compounds for the treatment of organ-specific autoimmunity.

## Results

### Engineering of pMHCIIs displaying an insulin B-chain epitope in three different registers

**Figure 1:**
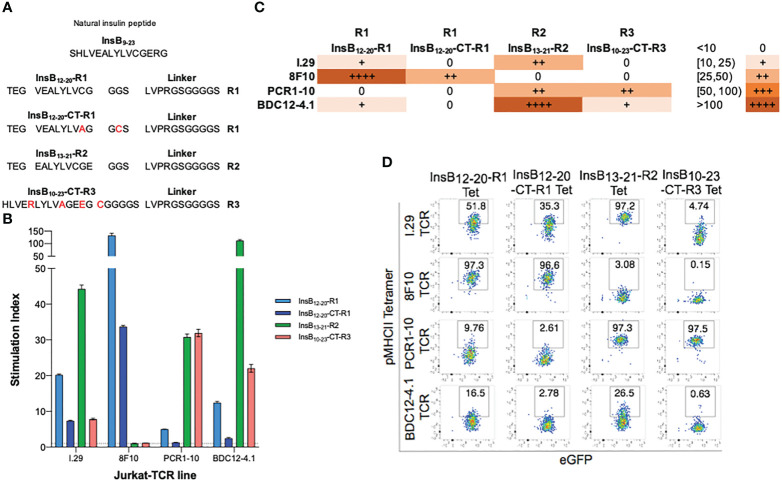
Reactivity patterns of four different InsulinB_9-23_/IA^g7^ pMHCIIs to reporter Jurkat cell lines expressing cognate TCRs. **(A)** Peptide-linker sequences of InsulinB_9-23_ bound to IA^g7^ on different registers. **(B)** NFAT-driven luciferase activity of four different TCR transfectants to plate-bound pMHCII monomers. Data are shown as the average ± S.E. from triplicate cultures. **(C)** Summary of the differential agonistic activity of the different pMHCIIs on the 4 different Jurkat cell lines from **(B)**. **(D)** Binding of the four pMHCII tetramers from **(A)** to the Jurkat cell lines used in **(B)**.

We first designed four different pMHCII-NPs displaying an insulin B-chain epitope bound to IA^g7^ in three different registers: 1) InsB_12-20_ peptide anchored in register #1 (type B) (12-20) (7), herein referred to as InsB_12-20_-R1 (this peptide cannot be displayed in registers 2 or 3 because it is truncated at position 20, deleting the pocket 9-anchoring E21 residue); 2) InsB_12-20_ peptide anchored to neighboring alpha chain residue *via* a Cys-trap to force binding of this epitope in register #1 (12-20) (this required the introduction of an additional C19A replacement in the InsB_10-20_ sequence), herein referred to as InsB_12-20_-CT-R1; 3) InsB_13-21_ anchored in register 2 (type A) (7) *via* an E21-pocket 9 interaction, herein referred to as InsB_13-21_-R2; and 4) InsB_10-23_ anchored in register 3 also *via* a Cys-trap, additionally containing mutations A14R and R22E to improve binding to p1 and p9, respectively, and C19A to allow the introduction of a Cys-trap, as in the peptide binding to register 2 above, based on Kappler’s design (8), herein referred to as InsB_10-23_-CT-R3. The peptide in the first three pMHCII designs carried a three amino acid extension both in the peptide’s amino terminal end (TEG) and the carboxyterminal end (GGS), as described in (9), and was tethered to the MHCII beta chain *via* a linker (LVPRGSGGGGS). The peptide in the fourth design carried a CGGGGS extension in its carboxy terminal end, immediately before the linker, as in (8) (**Figure 1A**).

We expressed these four different pMHCIIs in CHO cells and purified the corresponding monomers from the cell culture supernatants *via* sequential 6xHis and streptag affinity chromatography. We then tested the ability of pMHCII monomers to trigger TCR signaling in TCRαβ/mCD4-transduced TCRβ-deficient Jurkat/MA (JurMA) cells carrying a luciferase reporter driven by nuclear factor of activated T-cells (NFAT)-binding DNA (10), as described (1). We used constructs encoding the TCRαβ chains of the following insulin-reactive T-cell hybridomas: I.29, proposed to recognize InsB_9-23_ bound to IA^g7^ in registers 2 (7) or 3 (8); 8F10, proposed to recognize InsB_9-23_ bound to IA^g7^ in registers 1 (11) or 3 (8); PCR1-10, proposed to recognize InsB_9-23_ bound to IA^g7^ in registers 2 (7) or 3 (8); and BDC12-4.1, also proposed to recognize InsB_9-23_ bound to IA^g7^ in registers 2 (7) or 3 (8).

The pMHCIIs displaying InsB_12-20_-R1 and InsB_12-20_-CT-R1 almost exclusively stimulated the 8F10-JurMA cells, triggering only minor luciferase activity on I.29- and BDC12-4.1-JurMA cells (**Figures 1B, C**). The corresponding pMHCII tetramers bound primarily to the 8F10- and, to a lesser extent, I.29-JurMAs but not to the other two cell lines (**Figure 1D**). The 8F10-JurMA cells did not recognize the other two pMHCIIs (InsB_13-21_-R2 and InsB_10-23_-CT-R3) in both assays (luciferase activity and tetramer binding), indicating that InsB_12-20_ in our pMHCII molecular designs is indeed presented on a different register than InsB_13-21_-R2 and InsB_10-23_-CT-R3 (**Figures 1B–D**). In contrast, the pMHCII displaying InsB_10-23_-CT-R3 exclusively triggered luciferase activity on the PCR1-10-JurMA and, to a lesser extent, the BDC12-4.1-JurMA lines (**Figures 1B, C**). In agreement with these data, the corresponding pMHCII tetramer bound efficiently to the PCR1-10-JurMA, albeit not to the BDC12-4.1-JurMA (**Figure 1D**). The pMHCII displaying InsB_13-21_-R2 was primarily recognized by the BDC12-4.1-JurMA cells, but also triggered a lower response in the I.29- and PCR1-10-JurMA cells (**Figures 1B, C**). However, whereas the latter two cell lines bound tetramer efficiently, the former only did so weakly (**Figure 1D**). Taken together, these data indicate that pMHCIIs carrying InsB_12-20_-R1/InsB_12-20_-CT-R1, InsB_13-21_-R2 and InsB_10-23_-CT-R3 elicit three clearly distinct patterns of TCR reactivity, consistent with recognition of the epitope on three different registers, as expected.

### Insulin B-chain epitope-based pMHCII-NPs expand cognate CD4+ T-cells and trigger the formation of transcriptionally homogeneous pools of TR1-like cells

**Figure 2:**
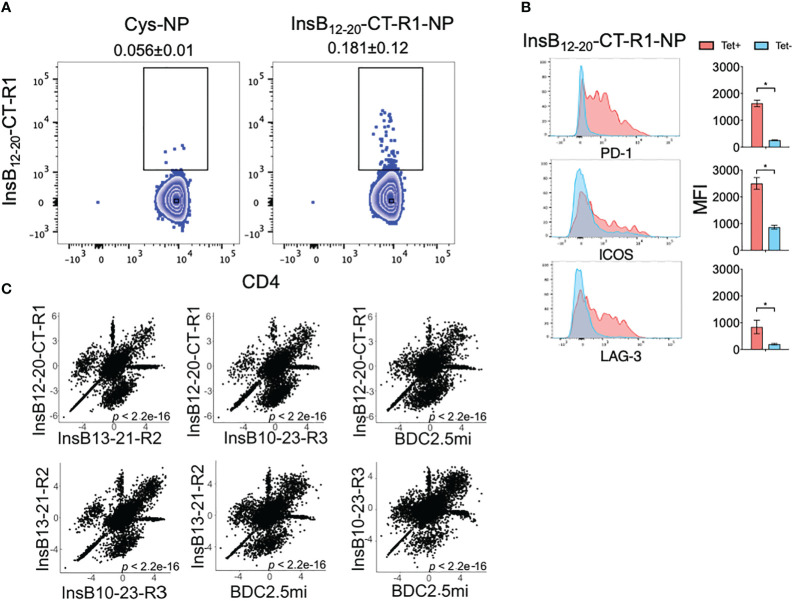
pMHCII-NP-induced TR1-like cell formation in pre-diabetic NOD mice *in vivo*. **(A)** Cognate tetramer staining profiles of splenic CD4+ T cells from NOD mice treated with NPs coated with pMHCIIs displaying the InsB_12-20_-CT-R1 epitope. Left panel, Cys-NP (control)-treated mice; Right panel, pMHCII-NP-treated mice. The average ± SEM values shown on top of each plot correspond to the percentages of Tet+ cells within the CD4+B220– gate (n=6 and 3 mice/NP type, respectively). **(B)** Flow cytometry staining profiles and average ± SEM mean fluorescence intensity (MFI) staining values for PD-1, ICOS and LAG-3 on tetramer+ versus tetramer– splenocytes. *, P<0.05 (Mann-Whitney U test). **(C)** Correlation between the levels of gene expression among tetramer+ cells isolated from mice treated with InsB_12-20_-CT-R1/IA^g7^-NP, InsB_13-21_-R2/IA^g7^-NP, InsB_10-23_-CT-R3/IA^g7^-NP and BDC2.5mi/IA^g7^-NP as compared to tetramer– cells (Tconv control) purified from InsB_12-20_-CT-R1/IA^g7^-NP-treated mice. The P value refers to the Pearson correlation coefficient for each comparison.

We have recently defined the transcriptional profile of the TR1-like cells that arise in NOD mice in response to BDC2.5mi/IA^g7^-NPs (2, 5). These CD4+ T-cells comprise transcriptionally and phenotypically homogeneous yet oligoclonal pools of cells. To investigate whether the response to NPs coated with insulin B-chain epitope-based pMHCII-NPs is alike, we first built pMHCII-based nanomedicines displaying the InsB_12-20_-CT-R1 pMHCII and tested its ability to trigger T-regulatory type 1 (TR1)-like cell formation and expansion *in vivo*. Treatment of 10 week-old pre-diabetic female NOD mice with this compound triggered the expansion of cognate CD4+ T-cells in spleen (as compared to NOD mice treated with control NPs) (**Figure 2A**). The splenic tetramer+ cells of these mice expressed the TR1 markers PD-1, ICOS and LAG-3 (2, 5), as expected (**Figure 2B**).

We next built pMHCII-NPs displaying the additional two Insulin B chain epitope-based pMHCIIs (InsB_13-21_-R2 and InsB_10-23_-CT-R3) and treated additional cohorts of mice with each of the three pMHCII-NP compounds (**Supplementary Figure 1A**). Since most of the tetramer+ cells arising in response to BDC2.5mi/IA^g7^-NPs upregulate both *Icos* and *Pdcd1* transcripts as compared to Tconv cells (5), and to minimize a potential contamination with Tconv cells, we sorted Tet+/PD-1+/ICOS+ cells from the pooled splenocytes of 5 mice/pMHCII-NP type (**Supplementary Figures 1B, C**), and used the SMARTseq2 approach to study cognate populations at the single-cell level. Although tetramer+ cells can also be found within the PD-1– and/or ICOS– gates of the different pMHCII specificities studied here (ICOS–/PD-1–: 0.02 ± 0.005%; ICOS+/PD-1–: 0.24 ± 0.04%; ICOS–/PD-1+: 1.15 ± 0.18%), most tetramer+ cells lie within the ICOS+/PD-1+ gate (1.7 ± 0.4%) (Average ± SE). The tetramer– CD4+ T-cells from the mice treated with Ins_12-20_-R1/IA^g7^-NP were used as T-conventional (Tconv) controls. As was the case for the TR1-like CD4+ T-cells arising in response to BDC2.5mi/IA^g7^-NPs (2, 5), the combined scRNAseq profiles of the tetramer+ T-cells arising in response to the three different pMHCII-NPs (as compared to the Tconv counterparts) showed significant upregulation of key TR1 signature genes such as the cytokine genes *Il10*, *Il21*, *Ifng*, the co-inhibitory receptor genes *Ctla4*, *Lag3*, *Tigit*, *Pdcd1*, the co-stimulator gene *Icos*, chemokine genes *Cxcr3* and *Cxcr5*, and the transcription factor genes *Bcl6*, *Maf*, *Ascl2*, *Nfil3* and *Tox2* and downregulation of several TR1-excluding genes, such as the cytokine receptor gene *Il7r*, the chemokine receptor gene *Ccr7*, and the transcription factor genes *Lef1* and *Klf2* (**Table 1**). Scatter plots of the log2FC values for differentially expressed genes in the tetramer+ cells arising in response to each pMHCII-NP type confirmed highly significant correlations among the scRNAseq profiles of the different tetramer specificities (P<10^-15^; >60% of all the genes in each comparison undergo similar levels of upregulation or downregulation as compared to Tconv cells) (**Figure 2C**). Thus, the tetramer+CD4+ T-cells that arise in response to Insulin B-chain (or BDC2.5mi) epitope-based pMHCII-NPs invariably express highly similar TR1-like transcriptional profiles.

**Table 1:** Differentially expressed genes in tetramer+ vs Tconv cells.

Gene	logFC	padj
***Pdcd1***	-3.420	1.714E-30
***Nt5e***	-3.141	7.311E-18
***Cxcr5***	-2.989	2.676E-22
***Il10***	-2.865	1.515E-05
***Nfil3***	-2.750	9.541E-07
***Ifng***	-2.704	2.460E-12
***Il21***	-2.703	2.030E-17
***Tigit***	-2.636	9.201E-24
***Lag3***	-2.507	2.028E-13
***Ascl2***	-2.445	1.649E-12
***Tox2***	-2.181	1.283E-16
***Bcl6***	-1.811	6.799E-21
***Maf***	-1.764	1.581E-14
***Rbpj***	-1.760	1.563E-05
***Cxcr3***	-1.697	6.764E-15
***Il4***	-1.686	1.377E-07
***Ctla4***	-1.665	1.041E-13
***Icos***	-1.210	1.231E-18
***Cd226***	-1.141	1.264E-13
***Il7r***	1.415	4.779E-19
***Ccr7***	1.549	1.008E-22
***Lef1***	1.607	2.711E-28
***Klf2***	2.667	6.605E-19
***Myc***	3.455	1.296E-15
***Sell***	4.367	1.082E-35

FC, fold change; Padj, adjusted P value.

**Figure 3:**
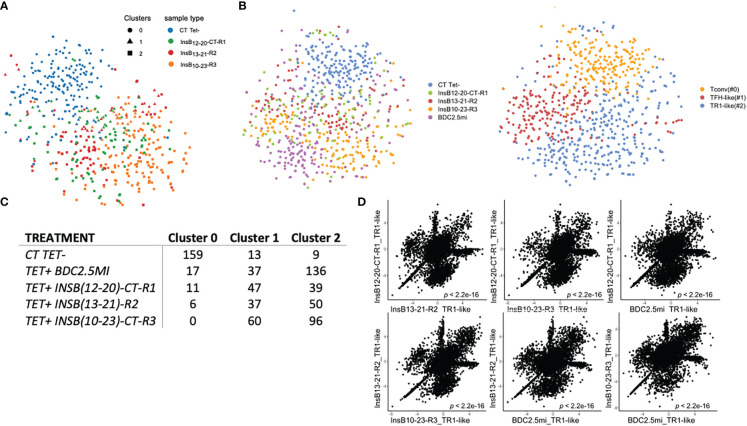
Single cell (sc) RNAseq analysis of tetramer+ CD4+ T-cells from pMHCII-NP-treated mice. **(A)** t-SNE plot of scRNAseq data for tetramer+ cells isolated from mice treated with InsB_12-20_-CT-R1/IA^g7^-, InsB_13-21_-R2/IA^g7^- or InsB_10-23_-CT-R3/IA^g7^-NPs as compared to tetramer– cells (control) purified from InsB_12-20_-CT-R1/IA^g7^-NP-treated mice. **(B)** t-SNE plots of scRNAseq data for cells from the mice in **(A)** plus tetramer+ cells from NOD mice treated with BDC2.5mi/IA^g7^-NPs. Left, cluster types; Right, pMHCII specificities. **(C)** Table depicting the number of cells belonging to each cell cluster as a function of tetramer-binding specificity. **(D)** Correlation between the levels of gene expression among TR1-like (cluster #2) cells from mice treated with InsB_12-20_-CT-R1/IA^g7^-NP, InsB_13-21_-R2/IA^g7^-NP, InsB_10-23_-CT-R3/IA^g7^-NP and BDC2.5mi/IA^g7^-NPs as compared to tetramer– cells (Tconv control) purified from InsB_12-20_-CT-R1/IA^g7^-NP-treated mice. The P value refers to the Pearson correlation coefficient for each comparison.

The tetramer+ T-cell pools arising in response to each of these three Insulin B-chain epitope-based pMHCII-NP types clustered into a major and a minor cluster (clusters 2 and 1, respectively). These clusters included most of the tetramer+ cells of each of the 3 treatment groups (InsB_12-20_-CT-R1/IA^g7^: 86/97 cells – 45% in cluster 2–; InsB_13-21_-R2/IA^g7^: 87/93 cells – 57% in cluster 2–; InsB_10-23_-CT-R3/IA^g7^: 156/156 cells – 62% in cluster 2–). Both clusters were clearly distinct from the major pool of cells (cluster 0) within the Tconv subset (88% of cells) (**Figures 3A, C**).

When compared to the tetramer– Tconv cell cluster 0, both tetramer+ cell clusters (#1 and #2) significantly downregulated the non-TR1 genes *Ccr7*, *Il7r* and *Lef1*, among others (negative logFC values under clusters #1 and #2 in **Table 2A**; comparisons in each column in **Tables 2A**, **C** refer to the genes in cluster X vs. all the other subclusters grouped together). The prevalent cluster of tetramer+ CD4+ T-cells (#2) displayed significant upregulation of many key TR1 markers as compared to clusters 0 and, to a lesser extent, cluster 1, including the cytokine genes *Il10*, *Il21*, *Ifng*, the co-inhibitory receptor genes *Ctla4*, *Tigit*, *Pdcd1*, and the transcription factor genes *Maf*, *Nfil3*, *Prdm1* and *Id2* (positive logFC values under cluster #2 in **Table 2B**), all previously found to be upregulated in bulk BDC2.5mi/IA^g7^-specific TR1-like cells (5). When compared to cluster #2, the minor cluster of tetramer+ CD4+ T-cells (cluster #1) displayed largely moderate, albeit significant, downregulation or upregulation of the cluster 2 signature genes (e.g., *Ifng* and *Cxcr3* or *Pdcd1* and *Prdm1*, respectively) (**Table 2B**), and it significantly upregulated TFH-associated genes, such as *Cxcr5*, *Cxcr4*, *Il4*, *Nfia* and *Tox2* (positive logFC values under cluster #1 in **Table 2C**). To further substantiate that each of the clusters identified herein correspond to true TFH- and TR1-like cell subsets, we compared the expression of genes specifically upregulated in the BDC2.5mi/IA^g7^ tetramer+ TR1 or TFH sub-pools (vs. all other tetramer+ cells) (to define TR1- or TFH-specific genes, respectively) (5) in the TFH-like and TR1-like cell clusters identified herein. Among the differentially expressed genes found in the Smartseq2 data reported here, 92% of the TFH-specific genes were specifically upregulated in cluster #1 (TFH-like, vs. Tconv cells), whereas 71% of the TR1-specific genes were specifically upregulated in cluster #2 (TR1-like, vs. Tconv cells) (P=1.072x10^-13^). Together, these data corroborate the invariable presence of a cognate intermediate TFH-like population that co-exists with the TR1 cells induced by the pMHCII-NP treatment (5).

**Table 2A:** Markers Cluster #0.

Gene	logFC Cluster 0	padj Cluster 0	logFC Cluster 1	padj Cluster 1	logFC Cluster 2	padj Cluster 2
***Sell***	**4.650**	**9.031E-38**	-3.029	1.087E-11	-4.763	9.651E-65
***Myc***	**3.630**	**3.446E-16**	-2.357	3.154E-12	-3.455	7.721E-46
***Klf2***	**2.134**	**2.310E-14**	-1.542	1.649E-13	-1.740	1.272E-42
***Lef1***	**1.985**	**3.084E-33**	-0.831	5.320E-12	-1.887	2.342E-61
***Ccr7***	**1.534**	**7.781E-24**	-1.521	1.132E-14	-1.048	2.616E-49
***Il7r***	**1.079**	**2.424E-18**	-0.873	3.349E-10	-0.722	1.656E-46

**Table 2B:** Markers Cluster #2.

Gene	logFC Cluster 0	padj Cluster 0	logFC Cluster 1	padj Cluster 1	logFC Cluster 2	padj Cluster 2
***Gzmb***	-3.103	1.000E+00	-2.850	1.000E+00	**3.385**	**2.770E-35**
***Ifng***	-2.839	1.352E-11	-0.908	9.528E-10	**2.188**	**3.103E-48**
***Cxcr3***	-1.572	6.947E-15	-2.290	6.303E-09	**2.067**	**6.833E-58**
***Il10***	-2.981	1.467E-05	-0.453	5.306E-07	**1.862**	**6.089E-35**
***Lag3***	-2.656	9.271E-13	-0.259	5.394E-08	**1.627**	**7.748E-44**
***Ascl2***	-2.595	1.197E-10	-0.185	3.077E-10	**1.551**	**1.105E-43**
***Ctla4***	-1.916	7.150E-16	-0.428	1.872E-06	**1.510**	**2.528E-45**
***Entpd1***	-1.001	5.632E-02	-1.771	3.824E-01	**1.476**	**3.433E-25**
***Nfil3***	-2.871	8.732E-07	0.004	5.136E-08	**1.445**	**5.346E-39**
***Tigit***	-2.798	4.135E-25	0.014	2.809E-06	**1.423**	**7.249E-64**
***Maf***	-1.751	5.187E-16	-0.261	3.296E-09	**1.324**	**1.760E-42**
***Il21***	-2.160	2.038E-15	0.033	4.015E-08	**1.254**	**4.237E-45**
***Prdm1***	-2.429	1.707E-02	0.190	1.000E+00	**1.193**	**1.674E-19**
***Pdcd1***	-2.890	1.097E-28	0.378	8.935E-08	**1.109**	**1.769E-43**
***Cd226***	-1.390	1.001E-13	-0.198	3.190E-07	**1.105**	**1.487E-41**
***Id2***	-0.797	8.639E-11	-0.800	3.675E-11	**1.025**	**6.765E-48**
***Tnfrsf4***	-1.432	9.392E-19	-0.014	3.538E-07	**1.004**	**1.579E-46**

**Table 2C:** Markers Cluster #1.

Gene	logFC Cluster 0	padj Cluster 0	logFC Cluster 1	padj Cluster 1	logFC Cluster 2	padj Cluster 2
***Il4***	-1.744	4.309E-07	**1.597**	**6.642E-11**	-0.297	8.967E-32
***Nfia***	-1.170	3.264E-02	**1.456**	**1.058E-04**	-0.384	2.602E-20
***Cxcr5***	-2.569	3.111E-22	**1.397**	**4.208E-14**	0.088	1.405E-39
***Tox2***	-2.045	9.971E-15	**1.234**	**4.646E-11**	0.148	2.897E-38
***Cxcr4***	-0.316	3.827E-13	**1.059**	**2.282E-09**	-0.558	6.557E-35

FC, fold change; Padj, adjusted P value. Data corresponding to the clusters 0, 2 and 1 in Tables A-C, respectively, are bolded.

To further probe the role of fine antigenic specificity in the outcome of these experiments, we compared the scRNAseq data from the tetramer+ cells arising in response to all three Insulin B-chain epitope-based pMHCII-NPs to those arising in response to BDC2.5mi/IA^g7^-NPs. As was the case for the three insulin B-chain-specific tetramer+ cell pools, most BDC2.5mi-Tet+ cells (91%) were found in clusters #2 (136/190 –72%–) and #1 (37/190 –19%–) (**Figures 3B, C**). Furthermore, scatter plots of the log2FC values for the differentially expressed genes of cluster #2 (TR1-like) cells confirmed highly significant correlations (P<10^-15^) among the gene expression profiles of the various epitope-specific TR1-like cells studied herein, indicating that NPs displaying four different T1D-relevant pMHCII types trigger the formation of similar, transcriptionally homogeneous pools of cognate TR1-like cells (**Figure 3D**). **Table 3** shows differences between the different populations for a selection of 106 TR1/Treg/TFH-relevant genes.

**Table 3:** Differentially expressed markers in treatment versus control groups.

Gene	Insulin treatments vs. control		BDC vs. Control (Ins Tet–)		BDC vs. Insulin treatments	
	logFC	padj	logFC	padj	logFC	padj
***Pdcd1***	3.42	1.7E-30	2.209	2.6E-25	-1.407	0.03062
***Nt5e***	3.141	7.3E-18	2.693	1.3E-24	-0.462	1
***Gzmb***	3.034	1	-0.044	1	-3.046	0.1178
***Cxcr5***	2.989	2.7E-22	-0.583	2.1E-18	-3.189	0.11131
***Il10***	2.865	1.5E-05	4.601	1.9E-43	2.665	5.4E-27
***Nfil3***	2.75	9.5E-07	3.617	3.3E-32	1.469	2.6E-13
***Ifng***	2.704	2.5E-12	3.233	1.4E-25	0.724	1
***Il21***	2.703	2E-17	2.156	3E-23	-0.673	1
***Tigit***	2.636	9.2E-24	3.068	8.6E-42	0.812	9.6E-08
***Lag3***	2.507	2E-13	2.291	1.6E-25	-0.186	1
***Ascl2***	2.445	1.6E-12	1.067	3.5E-13	-1.489	1
***Prdm1***	2.352	1	2.451	1.7E-08	0.125	1
***Il10Ra***	2.331	1	1.502	0.00015	-0.33	1
***Entpd1***	2.331	1	2.618	1.1E-09	0.368	1
***Tox2***	2.181	1.3E-16	1.747	2.7E-19	-0.618	1
***Tbx21***	1.825	1	1.798	0.33849	0.118	1
***Bcl6***	1.811	6.8E-21	0.952	3.8E-20	-0.821	1
***Maf***	1.764	1.6E-14	2.144	9.1E-26	0.347	1
***Rbpj***	1.76	1.6E-05	1.447	5.9E-09	-0.266	1
***Serpinb9***	1.752	1	ND	ND	-1.672	0.00162
***Cxcr3***	1.697	6.8E-15	0.456	1.3E-20	-1.295	1
***Il4***	1.686	1.4E-07	-0.204	0.00331	-1.732	1
***Ctla4***	1.665	1E-13	2.857	8.1E-49	1.253	5.1E-22
***Tgfbr1***	1.619	1	-0.088	1	-0.595	1
***Ebi3***	1.57	1	ND	ND	-1.53	0.00203
***S1pr2***	1.518	1	ND	ND	-1.454	0.00019
***Icos***	1.21	1.2E-18	1.676	4.9E-27	0.451	0.00121
***Cd226***	1.141	1.3E-13	0.658	5.4E-21	-0.479	1
***Nfia***	1.096	0.08018	1.429	8.7E-06	0.351	1
***Cbfa2t3***	1.059	1	-0.049	1	-1.08	1
***Stat3***	0.996	1.3E-13	0.994	4.9E-20	0.013	1
***Batf***	0.976	1.5E-15	0.649	2.6E-19	-0.394	1
***Id2***	0.899	3.6E-12	1.078	7.9E-22	0.166	1
***Cd28***	0.849	7.2E-15	1.283	1.1E-19	0.384	0.01741
***Tnfrsf4***	0.845	3.1E-13	1.734	2.6E-43	0.884	5.6E-14
***Irf1***	0.654	1E-14	0.795	1.4E-22	0.04	1
***Tnfrsf18***	0.629	2.1E-09	0.759	2.5E-15	0.124	1
***Sh2d1a***	0.514	3.1E-11	-0.461	4.4E-17	-0.909	1
***Il21r***	0.489	1.7E-12	0.863	7.3E-18	0.366	1
***Il27ra***	0.482	9.5E-08	-0.397	3.3E-19	-0.872	1
***Stat4***	0.458	6.8E-11	0.541	6.5E-17	0.038	1
***Tgfbr2***	0.441	1.1E-15	0.211	3E-18	-0.265	1
***Hmgb2***	0.368	8.9E-10	0.752	1.2E-16	0.354	1
***Elk4***	0.349	2.4E-11	0.45	3.8E-17	0.083	1
***Itk***	0.341	1.2E-13	0.549	1.2E-17	0.116	1
***Tcf7***	0.313	5.7E-14	0.171	2.4E-16	-0.155	1
***Cxcr4***	0.267	1.8E-13	2.022	2.2E-32	2.007	2.3E-44
***Cblb***	0.255	5.6E-11	1.342	7.7E-23	1.039	1.5E-08
***Id3***	0.237	5.9E-18	-1.576	8.8E-26	-1.678	1
***Cd40lg***	0.163	4.7E-12	-0.341	1.3E-19	-0.475	1
***Stat1***	-0.1	8.7E-09	-0.506	3.3E-11	-0.424	1
***Selplg***	-0.368	2.1E-10	-1.124	5.5E-18	-0.762	1
***Rora***	-0.401	8.2E-08	0.459	1.7E-16	0.887	0.00035
***Foxp1***	-0.404	1.3E-09	-0.487	1.3E-15	-0.098	1
***Il2ra***	-0.627	5.8E-07	-0.93	1.6E-09	-0.227	1
***Foxp3***	-0.692	5.5E-11	-3.151	5.6E-12	-1.972	1
***Bmyc***	-0.723	6.4E-10	-1.812	1.2E-11	-0.826	1
***S1pr1***	-0.87	1.7E-20	-0.293	6.1E-09	0.604	0.36342
***Il7r***	-1.415	4.8E-19	-1.002	7.5E-22	0.349	1
***Ccr7***	-1.549	1E-22	0.249	5.4E-13	1.737	2.5E-19
***Lef1***	-1.607	2.7E-28	-1.505	3.8E-27	0.161	1
***Klf2***	-2.667	6.6E-19	-0.324	1.3E-13	2.208	5.4E-10
***Myc***	-3.455	1.3E-15	-1.981	2.4E-11	1.547	0.00365
***Sell***	-4.367	1.1E-35	-1.197	5E-25	3.08	1.4E-08

FC, fold change; Padj, adjusted P value; ND, not detected.

Thus, pMHCII-NP therapy invariably triggers the formation and expansion of transcriptionally homogeneous pools of cognate TR1 cells that co-exist with smaller pools of cognate TFH-like cells, consistent with a lineage relationship between the two.

### Oligoclonal nature of insulin epitope-specific pMHCII-NP-induced TR1 cells

**Figure 4:**
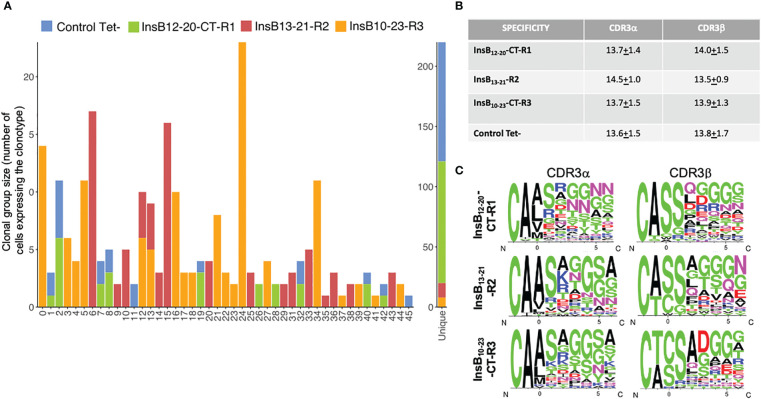
Clonotypic diversity of the tetramer+ vs tetramer- CD4+ T-cells arising in pMHCII-NP-treated mice. **(A)** Distribution of clonal sizes and TCR specificity. Data correspond to number of cells expressing each of the 45 different TCRαβ pairs that were found in more than one cell, or the ~300 that were found only once (labeled as “unique” in the plot). **(B)** Average length in amino acids (± SEM) of the CDR3α or CDR3β sequences of the TCRs expressed by the tetramer+ cells arising in response to treatment with the three different pMHCII-based nanomedicines. **(C)** Schematic representation of the percentage of prevalent amino acids at specific positions at the N terminus of CDR3 sequences (first 6 residues) for each pMHCII specificity normalized against the frequency of each residue at each position. Numbering of the CDR3 residues follows the nomenclature of Gioia et al., 2019 (12).

We next asked if the various pools of insulin B-chain epitope-specific TR1-like CD4+ T-cells arose from one or multiple cognate T-cell clones. We analyzed the TCRαβ rearrangements expressed by the single cells analyzed above using TraCeR (12); cells containing identical combinations of V(D)Jαβ elements and junctional sequences were considered to belong to a single clonotype. As was the case for the BDC2.5mi/IA^g7^-specific TR1-like cells induced by the corresponding pMHCII-based nanomedicines (5), the tetramer+ T-cell pool arising in response to InsB_12-20_-CT-R1/IA^g7^ was oligoclonal, with 114 different TCRαβ pairs out of 144 different clonotypes identified (**Figure 4A** and **Supplementary Data Sheet #1**). None of these TCRαβ pairs were found in the tetramer+ T-cell pools arising in mice treated with pMHCII-NPs displaying the other two insulin registers. However, some of these repeated clonotypes (n=10) could also be found in the Tconv (tetramer–) T-cell pool sorted from the same mice. The presence of such clonotypes in both the tetramer+ and tetramer– gates suggests that they bind InsB_12-20_-CT-R1/IA^g7^ tetramer with low avidity and, as a result, near the threshold of detection by flow cytometry. This is not entirely surprising, given the low sensitivity of pMHCII tetramer staining, particularly for autoreactive T cell specificities (13, 14). We note that all the other clonotypes found in the tetramer– pool, except two (clonal groups 11 and 45, repeated three times and twice, respectively), were unique (i.e., not repeated), as expected, thus suggesting that the 10 repeated clonotypes found in both the tetramer+ and tetramer– pools are not due to a contamination of the former by cells from the latter (i.e., as a result of using a rather inclusive gate for sorting; see **Supplementary Figure 1**). The tetramer+ CD4+ T-cell pools arising in response to NPs displaying InsB_13-21_-R2/IA^g7^ and InsB_10-23_-CT-R3/IA^g7^ pMHCIIs were also oligoclonal, albeit significantly less diverse than those arising in InsB_12-20_-CT-R1/IA^g7^-NP-treated mice, containing only 13/94 and 9/155 unique (non-duplicated) TCR sequences, respectively). These two populations shared 2 clonotypes (**Figure 4A** and **Supplementary Data Sheet #1**), possibly due to register shifting of the longer InsB_10-23_-CT-R3 epitope, such that it can also be displayed on register 2. The oligoclonality of the latter two InsB register specificities was accompanied by skewed Vα, Jα and Vβ element usage (e.g. TRAV5D-4, TRAJ18 and TRBV1: n=52/129, 24/129 and 22/110, respectively, for InsB_13-21_-R2/IA^g7^-specific T cells, and n= 77/181, 48/181 and 98/146, respectively for InsB_10-23_-CT-R3/IA^g7^-specific T cells) as compared to InsB_12-20_-CT-R1/IA^g7^-specific T cells (n=27/154, 14/154 and 7/140 respectively) or tetramer– cells (n=14/131, 10/131 and 12/117, respectively) (P<0.0001) (**Supplementary Figure 2**). There were no obvious differences in the lengths of the CDR3α or CDR3β regions of the TCRs expressed by each specificity (**Figure 4B** and **Supplementary Data Sheet #2**).

It has been shown that the TCRs expressed by the Ins_12-20_/IA^g7^ reactive CD4+ T-cells isolated from the islets and pancreatic lymph nodes of young pre-diabetic NOD mice have a negatively charged residue at P2 or P3 of the CDR3β region, to overcome the lack of a negatively amino acid at the peptide’s P9 position (15). Although some of the pMHCII-NP-induced InsB_12-20_-CT-R1/IA^g7^-specific TR1-like cells studied here had this feature (i.e., 37.2% of clonotypes carried a D or E at P2 or P3, as compared to 10.1% of the InsB_13-21_-R2/IA^g7^-reactive clonotypes), this was also seen in 51.2% of InsB_10-23_-CT-R3/IA^g7^ pMHCII-reactive clonotypes, which recognize a peptide (InsB_10-23_-CT-R3) carrying a negatively charged residue at P9 (**Figure 4C** and **Supplementary Data Sheets #2, #3**).

Collectively, these data demonstrate that pMHCII-NP therapy triggers the differentiation of oligoclonal subsets of cognate CD4+ T-cells into populations of TR1-like cells that have homogeneous gene expression profiles but clearly distinct TCRαβ sequence patterns.

### Equivalent distribution of identical clonotypes within the TFH and TR1 tetramer+ sub-clusters

**Figure 5:**
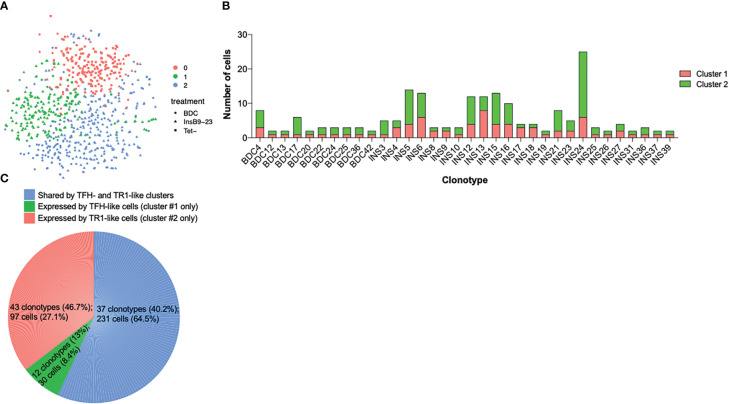
Cluster distribution of repeated clonotypic TCRαβ pairs within tetramer+ cells of pMHCII-NP-treated mice. **(A)** Seurat clustering analysis of SMARTseq2-based scRNAseq data for sorted tetramer+ and tetramer– cells from NOD mice treated with BDC2.5mi/IA^g7^- or InsB_9-23_/IA^g7^-NPs (from n=5 and 15 mice, respectively). Most tetramer+ cells (63.6%) are found in cluster #2 (TR1-like). **(B)** Sub-cluster distribution of each of the repeated clonotypic TCRαβ pairs (found in more than one cell) for tetramer+ cells from NOD mice treated with BDC2.5mi/IA^g7^- or InsB_9-23_/IA^g7^-NPs. **(C)** Pie chart displaying the percentage of repeated clonotypes shared by clusters #1 and #2 or expressed only by clusters #1 or #2 among all clonotypes identified, as well as the number of cells expressing these clonotypes among all cells analyzed.

We next ascertained the distribution of the repeated clonotypic TCRαβ pairs (i.e. expressed by more than one cell) within the two sub-clusters that are contained within the tetramer+ pools. Remarkably, many of the BDC2.5mi- and insulin-register-specific clonotypes identified herein were found in both cluster #1 (TFH) and cluster #2 (TR1), with distributions that paralleled the relative size of the corresponding clusters (**Figures 5A, B** and **Supplementary Data Sheet #4**). **Figure 5C** provides a pie chart displaying the percentage of repeated clonotypes shared by clusters #1 and #2 or expressed only by clusters #1 or #2 among all clonotypes identified, as well as the number of cells expressing these clonotypes among all cells analyzed. Thus, clusters #1 and #2 correspond to identical clonotypes at two distinct differentiation states (a TFH-like and a TR1-like), further substantiating a direct lineage relationship between these two cell types.

### Insulin B-chain register-specificity of representative TCRαβ pairs

We next sought to verify the specificity of clonotypes that expanded in response to the three different insulin epitope-specific pMHCII-NP types described above. We focused on TCRαβ pairs within each TR1 cell pool that were most frequent and not found in the negative control population (**Supplementary Data Sheet #6**; the corresponding clonotypes are also identified in **Supplementary Data Sheets #1-3, #5**). For InsB_12-20_-CT-R1/IA^g7^ we used clonotype #26 (NXT-258, repeated 2 times out of 144 sequenced/productive TCRs). For InsB_13-21_-R2/IA^g7^ we chose the most prevalent clonotype (#6 –NXT-10–, 18 copies out of 94 sequenced/productive TCRs). For InsB_10-23_-CT-R3/IA^g7^ we chose a prevalent clonotype (#0, –NXT-297–, 15 of 155 sequenced/productive TCRs) that had a negative charged residue (Asp) at position 2 of the CDR3β. We used these three recombinant TCRs to build the corresponding reporter JurMA cell lines [expressing the TCRαβ along with the mCD4 co-receptor and an NFAT-luciferase transgene (1)].

**Figure 6:**
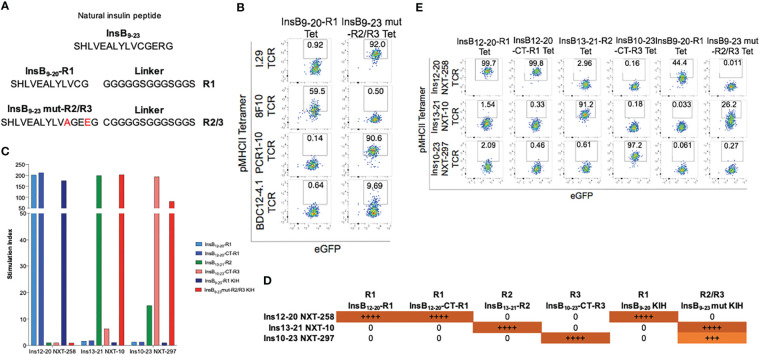
Reactivity patterns of two additional Insulin B_9-23_/IA^g7^ pMHCII designs to reporter Jurkat cell lines expressing the TCRs studied in **Figure 1** or TCRs cloned from tetramer+ cells arising in pMHCII-NP-treated NOD mice. **(A)** Peptide-linker sequences of two novel pMHCII designs, displaying Insulin B_9-23_ bound to IA^g7^ on the R1 or R2/R3 registers. **(B)** Binding of the two pMHCII tetramers from A to the Jurkat cell lines used in **Figure 1B**, **(C)** NFAT-driven luciferase activity in Jurkat cell lines expressing TCRαβ pairs cloned from pMHCII-NP-induced tetramer+ cells, in response to the six different pMHCII monomers described in **Figures 1A**, **(A)**. **(D)** Summary of the differential agonistic activity of the various pMHCIIs against the 3 Jurkat cell lines expressing TCRαβ pairs cloned from pMHCII-NP-induced TR1 cells. **(E)** Binding of the six pMHCII tetramers to the Jurkat cell lines used in **(C)**.

We then tested the ability of InsB_12-20_-CT-R1/IA^g7^, InsB_13-21_-R2/IA^g7^ and InsB_10-23_-CT-R3/IA^g7^ pMHCII tetramers and monomers described on **Figure 1A** to bind to, and trigger TCR signaling on these three new JurMA cell lines, respectively. We also tested the responsiveness of these three new cell lines to two new pMHCIIs displaying the native insulin epitopes bound to IA^g7^ in the R1 and R3 registers, without N- or C-terminal amino acid additions: a pMHCII containing the InsB_9-20_ anchored in R1 (Leader-SHLVEALYLVCG-flexible linker GGGGGSGGGSGGS; hereinafter referred to as InsB_9-20_-R1 KIH), and a pMHCII with InsB_9-23_ carrying a pocket 9-anchoring R22E mutation (8) (referred to as InsB_9-23_mut-R2/R3 KIH) (**Figure 6A**). This last pMHCII was designed to allow the binding of the peptide in R2 and/or R3. These two new compounds were engineered using a knob-into-hole-based heterodimerization approach that enables the production of these molecules at very high yields (14). As expected, whereas the InsB_9-20_-R1 KIH-based pMHCII teramer only bound to the 8F10-JurMA, the InsB_9-23_mut-R2/R3 KIH tetramer bound to I.29- and PCR1-10-JurMAs and weakly to the BDC12-4.1-JurMA line (**Figure 6B**).

As shown in **Figures 6C–E**, each cloned TCR recognized exclusively the cognate pMHCII used in the treatment. The InsB_12-20_-specific TCR responded (**Figures 6C, D**) and bound (**Figure 6E**) to InsB_12-20_-R1, InsB_12-20_-CT-R1 and InsB_9-20_-R1 pMHCII monomers and tetramers, respectively; the InsB_13-21_-R2-derived TCR responded (**Figures 6C, D**) and bound (**Figure 6E**) to InsB_13-21_-R2 pMHCIIs as well as the InsB_9-23_mut-R2/R3 KIH pMHCII. Lastly, the InsB_10-23_-CT-R3-specific TCR recognized the InsB_10-23_-CT-R3 and the InsB_9-23_mut-R2/R3 KIH pMHCIIs, although it only bound to the InsB_10-23_-CT-R3 tetramer, suggesting that the InsB_9-23_mut-R2/R3 KIH-based tetramer primarily displays the peptide in the R2 register.

Together, the above results demonstrate that the insulin B-chain-specific TFH/TR1 clonotypes arising *in vivo* in response to pMHCII-NPs displaying a single epitope on three different registers contain register-specific TCRs. Furthermore, the novel TCR clonotypes described herein have significantly higher avidity than their published counterparts and are exquisitely register-specific.

### Insulin epitope-based pMHCII-NPs blunt the progression of hyperglycemia in spontaneously diabetic NOD mice

**Figure 7:**
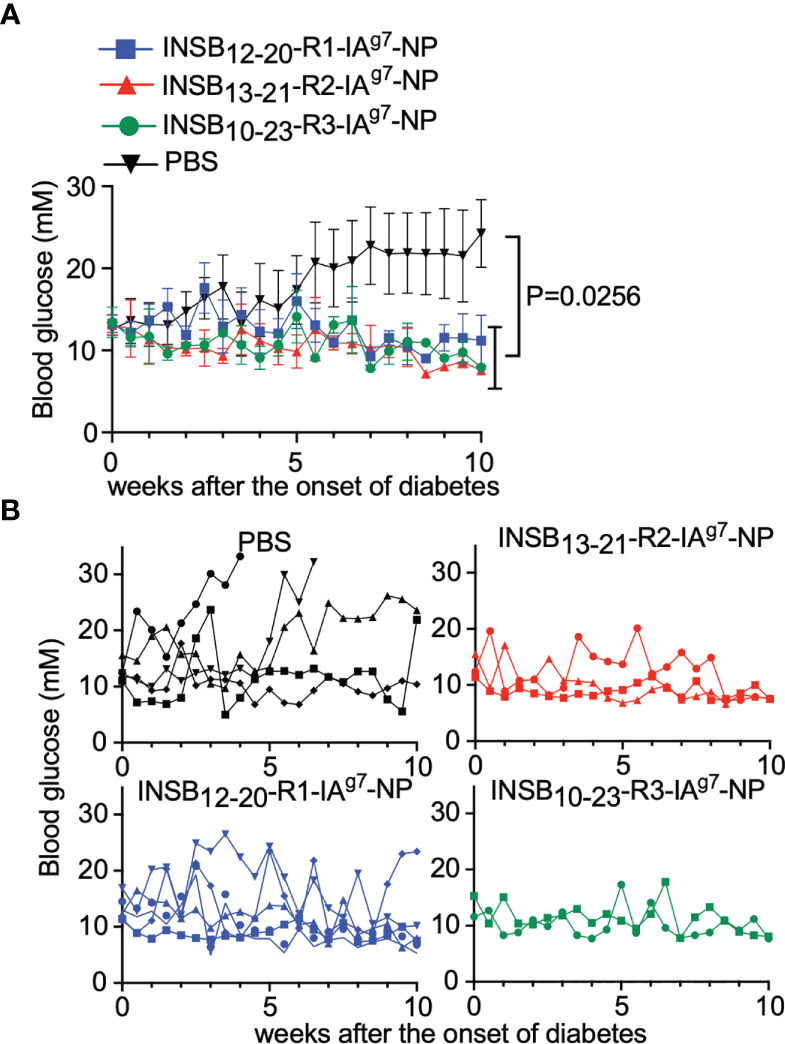
Reversal of hyperglycemia by insulin_9-23_/IA^g7^-NPs in newly diabetic NOD mice. **(A, B)** Evolution of average **(A)** and individual **(B)** blood glucose levels in diabetic female NOD mice treated with vehicle (n=5) or InsB_12-20_-CT-R1/IA^g7^-, InsB_13-21_-R2/IA^g7^- or InsB_10-23_-CT-R3/IA^g7^-NPs (n=5, 3 and 2, respectively). Data were compared *via* two-way ANOVA.

We next investigated the therapeutic properties of the above three insulin epitope-based pMHCII-NPs to blunt the progression of hyperglycemia in spontaneously diabetic NOD mice. Female NOD mice displaying hyperglycemia (>11mM blood glucose) were treated with vehicle (PBS) or each of the three pMHCII-NP types. Three mice that died shortly after initiation of treatment were excluded from analysis. Whereas 4/5 vehicle-treated mice progressed to overt hyperglycemia, 9/10 of the mice treated with these nanomedicines attained blood glucose levels <11mM within 10 weeks (**Figure 7**).

## Discussion

Insulin is a major CD4+ T-cell autoantigen in autoimmune diabetes, both in mice and humans (16, 17). In NOD mice, the anti-insulin CD4+ T-cell response is highly focused on B-chain residues 9-23 (SHLVEALYLVCGERG). Mohan et al. described two sets of CD4+ T-cell specificities that recognize this epitope in the context of IA^g7^, but on different binding registers (7, 18). One of these T-cell subsets, referred to as ‘type A’, targets the prevalent, most stable epitope that arises from the processing of the insulin molecule by professional APCs (Insulin B-chain residues 13-21 bound to IA^g7^ on register 2). The second subset, ‘type B’, exclusively targets a form of the epitope that is generated exclusively within the secretory granules of pancreatic beta cells and is then directly captured and presented by IA^g7^ on APCs, bypassing the APC antigen processing machinery (Insulin B-chain residues 12-20 bound to IA^g7^ on register 1) (7, 18). Unlike CD4+ T-cells targeting InsB_13-21_/IA^g7^, those targeting InsB_12-20_/IA^g7^ would bypass central tolerance. As a result, it has been proposed that this T-cell specificity represents the most prevalent and primary component of the diabetogenic anti-insulin T-cell response. In contrast, Kappler and colleagues have argued that InsB_9-23_ can also bind to IA^g7^ on two additional registers (8): registers #3 (InsB_14-22_: EALYLVCGER) and #4 (InsB_15-23_: ALYLVCGERG). Of these, register #3 is predicted to be the least favored of the four, because it would place an overtly conflicting positively charged arginine side chain in the positively charged pocket 9. Upon optimizing the p1 and p9 positions of the peptide for binding to IA^g7^ in different binding registers and by forcing the presentation of the epitope on register #3 by introduction of a cys-trap, these authors found that most insulin B-chain-specific T-cell hybridomas tested recognized the peptide bound on register #3 (BDC12-4.1, AS91, AS150, I.29). More recently, using soluble peptides and IA^g7^-tethered peptides carrying additional amino acid substitutions, these authors found that type A (register #2) and type B (register #1) T-cell hybridomas differentially respond to two register #3-binding epitope mutants (19). Based on these observations, Kappler et al. have proposed that these epitope variants might be generated *in vivo* by transpeptidation (19), i.e. as hybrid insulin peptides (HIPs) (20).

Here, we have taken advantage of the ability of pMHCII-NPs to trigger the conversion of cognate pMHCII-experienced CD4+ T-cells into TR1-like cells, in association with systemic expansion, to explore the transcriptional properties, clonality and fine antigenic specificity of pMHCII-NP-induced TR1-like cells. Our initial *in vitro* experiments, exploring the reactivity of the type A I.29, PCR1-10 and BDC12-4.1 and type B 8F10 TCRs (in a Jurkat cell-based reporter assay) to recombinant pMHCIIs displaying the prevalent InsB epitope on registers #1, #2 or #3, were more consistent with Unanue’s observations. Specifically, whereas the three type A TCRs that we tested primarily recognized the InsB_13-21_/IA^g7^-R2 complex (found to recognize the 8E9E register #3-binding variant by Wang et al.), the type B 8F10 TCR exclusively recognized the Ins_12-20_/IA^g7^-R1 pMHC (found to recognize the 8G9E register #3-binding variant by Wang et al.). Only the type A PCR1-10 TCR recognized Wang et al.’s cys-trapped InsB_10-23_/IA^g7^ pMHCII (the 8E9E variant equivalent), albeit to the same extent as the I.29 TCR, and significantly less than the BDC12-4.1 TCR. Collectively, these results supported the view that most, but certainly not all InsB_9-23_/IA^g7^-specific CD4+ T-cells recognize the epitope on registers #1 or #2.

By carrying extensive transcriptomic studies of BDC2.5mi/IA^g7^-NP-induced CD4+ T-cells, we have shown that such cells co-express many of the markers previously ascribed to different subsets of TR1-like cells (21–23). These previous studies, focused on BDC2.5mi/IA^g7^-specific TR1-like cells, had suggested but did not unambiguously establish that pMHCII-NP-induced TR1-like cell pools are a collection of transcriptionally homogeneous pools of clones. It also remained unclear whether the outcome of therapy with these compounds varies as a function of the pMHCII specificity, peptide-binding register or engineering design. Our results clearly show that the TR1-like cells arising in response to different pMHCII-NP types are transcriptionally similar regardless of the pMHCII type used or the MHCII-binding register *via* which the epitope is presented. Specifically, the scRNAseq profiles of the tetramer+ CD4+ T-cells arising in response to three insulin and the BDC2.5mi epitope-based pMHCII-NPs vs. their Tconv counterparts showed significant upregulation of key TR1 signature genes, such as the cytokine genes *Il10*, *Il21*, *Ifng*; the co-inhibitory receptor genes *Ctla4*, *Lag3*, *Tigit*, *Pdcd1*; the co-stimulator gene *Icos*; the chemokine receptor gene *Cxcr3*; and the transcription factor genes *Maf* and *Nfil3*. Importantly, the homogeneous transcriptional landscape of the TR1-like cells arising in response to the four different pMHCII-NP types tested herein mirrored a striking similarity with TFH cells, which we have recently identified as a precursor of pMHCII-NP-induced TR1-like cells and co-exist with transitional and terminally-differentiated TR1 cell sub-clusters within the pMHCII-NP-induced tetramer+ cell pools (5). Pseudotime analyses of scRNAseq data from BDC2.5mi/IA^g7^-NP-induced tetramer+ cells were consistent with a TFH-to-TR1 direction of transdifferentiation (5). This was further substantiated by demonstrating that BDC2.5mi/IA^g7^-NPs can trigger cognate TR1 cell formation in TFH-transfused immunodeficient hosts, and that T-cell-specific deletion of *Bcl6* or *Irf4* blunts both, BDC2.5mi/IA^g7^-NP-induced TFH expansion and TR1 formation. In contrast, deletion of *Prdm1* selectively blunts the TFH-to-TR1 conversion, demonstrating that generation of TR1 cells by pMHCII-NPs is preceded by cognate TFH cell expansion. We further showed that *Bcl6* and *Prdm1* are also necessary for anti-CD3 mAb-induced TR1 formation (5). In fact, comparison of the cluster of anti-CD3 mAb-induced TR1-like cells most closely related to the TR1 cells induced by the various pMHCII-NP specificities studied herein (5) reveal a highly significant statistical correlation in differential gene expression (**Supplementary Figure 3**). Thus, although the “TR1-like” cell pools induced by different methods (i.e., pMHCII-NPs or anti-CD3) are heterogeneous, containing precursor and transitional subsets, the terminally differentiated TR1 cells elicited by these different approaches are transcriptionally similar. Additional experimentation demonstrated that whereas the TFH-like cells arising in these studies, including those arising in mice carrying *Prdm1*-deficient T-cells, have TFH-like function but not immunoregulatory properties, their IL-10-producing TR1-like counterparts have profound immunoregulatory properties in an experimental encephalomyelitis (EAE) model (5). Importantly, the scRNAseq studies shown herein consistently identify a TFH-like cell sub-cluster in the Tet+ pools arising in response to all three nanomedicines, consistent with an invariable TFH origin for pMHCII-NP-induced TR1-like cells. In fact, the TCRαβ pairs that were found in more than one cell were found in both the TFH-like and the TR1 sub-clusters, strongly supporting a direct lineage relationship between these two cell types.

Our TCR sequencing data showed that the cognate T-cell pools that arise in response to the three different pMHCII-NP types studied here are oligoclonal, indicating that these compounds simultaneously re-program an oligoclonal repertoire of cognate pMHCII-specific T-cell precursors. The TCRαβ repertoire displayed by the tetramer+ CD4+ T-cell pools arising in response to NPs displaying InsB_13-21_-R2/IA^g7^ and InsB_10-23_-CT-R3/IA^g7^ pMHCIIs was less diverse than that arising in InsB_12-20_-CT-R1/IA^g7^-NP-treated mice. Since this is associated with skewed Vα, Jα and Vβ element usage (**Supplementary Figure 2**), it is likely that reactivity against these two insulin epitope/IA^g7^ complexes is favored by expression of certain VαJα/VβDβJβ rearrangements. Like the TCRs of InsB_12-20_/IA^g7^ reactive CD4+ T-cells isolated from the islets and pancreatic lymph nodes of young pre-diabetic NOD mice, which are enriched in Asp or Glu at P2 or P3 of the CDR3β region (24), 37.2% of the pMHCII-NP-induced InsB_12-20_-R1/IA^g7^-specific clonotypes, but not their Ins_13-21_/IA^g7^-R2-specific counterparts (10.1%), also exhibited this feature (as in Gioia et al.). However, over half of the InsB_10-23_-R3/IA^g7^-NP-induced clonotypes (51.2%) also carried a prevalent Asp at CDR3β’s P3 despite the fact that the peptide carried an R9E substitution at P9, introduced to enhance IA^g7^ binding. Thus, while the presence of a positive charge around P9 might favor recognition of the peptide by such Asp/Glu-containing CDR3β (15, 24), our data indicate that such CDR3β’s can also recognize insulin registers with a negative charge at this position.

Additional work with Jurkat cells expressing representative TCRs from the corresponding tetramer+ T-cell pools demonstrated that the clonotypes arising in response to each of the three nanomedicines tested were highly specific and non-crossreactive. Furthermore, these studies show that the peripheral InsB_9-23_/IA^g7^-specific T-cell repertoire does indeed contain specificities capable of exclusively recognizing InsB_10-23_-R3/IA^g7^ pMHCIIs. Thus, Jurkat cells expressing a representative TCR from tetramer+ T-cells induced in response to InsB_10-23_-R3/IA^g7^-NPs exclusively recognized InsB_10-23_-R3/IA^g7^ but not their InsB_13-21_-R2/IA^g7^ and InsB_12-20_-R1/IA^g7^ counterparts. Collectively, these observations indicate that the peripheral CD4+ T-cell repertoire in NOD mice harbors clones recognizing the prevalent InsB chain epitope on each of the three registers, and that these T-cells are inherently register-specific.

Lastly, we demonstrate that the three Insulin epitope-based nanomedicines tested herein have anti-diabetogenic activity *in vivo*. We do not know if the enlarged splenic tetramer+ cell pools arising in response to these insulin-epitope-based compounds are also enlarged within the islet cell infiltrates, but data from BDC2.5mi/IA^g7^-NP-treated mice suggest that this is likely the case. Specifically, the islet-associated CD4+ T-cells from BDC2.5mi/IA^g7^-NP-treated mice were found to harbor significantly increased percentages of BDC2.5mi/IA^g7^ tetramer^+^ T-cells than mice treated with control NPs, and the islet-associated tetramer^+^ cells from these mice were enriched for the TR1 sub-cluster found in the splenic tetramer^+^ T cell pool, at the expense of its transitional TR1-like and TFH cell counterparts, consistent with an increased tropism for sites of inflammation (5). Although the small number of mice tested with each pMHCII-NP type precludes definitive determination of any significant differences in therapeutic activity among the three different insulin epitope-based pMHCII-NP types, the outcome of these studies is similar to that reported for three other T1D-relevant pMHCII-NP types (2). Similar observations have been made for pMHCII-NPs targeting central nervous system and/or liver autoimmune diseases (2–4, 6). In all cases, T cell autoantigenic experience and local expression of the autoantigen that encodes the epitope displayed on the pMHCII-NP compound, rather than nature of the autoantigen, are key (2–4, 6). Recognition of endogenous autoantigen-loaded APCs by the pMHCII-NP-induced TR1 cells enables the productive activation of the latter, leading to local secretion of regulatory cytokines, suppression of autoantigen presentation and bystander immunoregulation (2–4, 6). Taken together, these data suggest that pMHCII-NPs have similar therapeutic properties regardless of the peripheral frequency of the target T cells. Collectively, our data suggest that pMHCII-NPs invariably trigger the terminal differentiation of multiple antigen-specific TFH clones into TR1-like cells with a defined transcriptional program, thus further supporting the translational significance of these compounds for the treatment of organ-specific autoimmunity, including T1D.

## Methods

### Mice

NOD/Lt mice were purchased from the Jackson Lab (Bar Harbor, ME). Mice were housed in specific pathogen-free facilities at the Cumming School of Medicine at the University of Calgary or at the Universitat de Barcelona. The experiments described herein were approved by the University of Calgary and Universitat de Barcelona Animal Care Committees.

### pMHCII production

Recombinant pMHC class II were produced in CHO-S cells transduced with lentiviruses (Vector Builder, Chicago, IL) encoding peptide-MHCα and MHCβ chains and IRES-CFP and IRES-EGFP cassettes, respectively. Unless indicated otherwise, the pMHCIIs used herein included a c-jun/c-fos heterodimerization domain. Where indicated, the c-jun/c-fos domains were replaced with IgG- knob and hole, to produce knob-into-hole (KIH)-based pMHCIIs, as described (14). To express the various pMHCs, transduced CHO cells were grown in 2 L baffled flasks (Nalgene, Thermo Fisher Scientific, Waltham, MA, USA) at 125 rpm, 5% CO_2_ and 37°C. Basal medium was Power-CHO-2 (Lonza, Basel, Switzerland) supplemented with 8 mM Glutamine (Cultek, Madrid, Spain) and Gentamicine Sulfate (0.25 mg/mL) (Lonza). The cultures were started in a volume of 400 ml of basal medium at a cell density of 350,000-400,000 cells/mL and were supplemented with Cell Boost 7a (Hyclone) at 3% v/v and Cell Boost 7b (Hyclone, GE Healthcare, Chicago, IL, USA) at 0.3% v/v on days 0, 3, 4, 5, 6, 8, 9 and 10. Temperature shift to 34°C was done when cell densities reached 5-7x10^6^ cells/mL. Additional Glutamine was added on day 7, to 2 mM. Glucose was added to 4.5 g/L when levels dropped below 3.5 g/L. Cells were harvested on Day 14 or when viability fell below 60%. The secreted proteins were purified by sequential affinity chromatography on nickel and strep-tactin columns (for c-fos/c-jun-based pMHCII) or protein A/G columns (for KIH-based pMHCII) and used for NP coating or biotinylated *in vitro* (for peptide-tethered pMHCII) to produce pMHC tetramers using fluorochrome-conjugated streptavidin.

### pMHC tetramers

Phycoerythrin (PE)-conjugated tetramers were prepared using biotinylated pMHCII monomers and used to stain peripheral T-cells as described (25, 26). Briefly, pMHCII monomers were subjected to biotinylation using Biotin ligase (Avidity, Aurora, CO, USA) following the supplier’s protocols, followed by ion exchange chromatography using an AKTA FPLC system (GE Healthcare, Chicago, IL, USA). The final product was verified by denaturing SDS-PAGE. Tetramers were generated by adding PE-conjugated streptavidin (Rockland Immunochemicals, Limerick, PA, USA) at a 4:1 molar ratio.

### Antibodies, peptides, flow cytometry

All antibodies were purchased from the indicated commercial suppliers. To stain mononuclear cell suspensions from mice, splenic CD4+ T-cells were incubated with anti-CD16/CD32 mAb (2.4G2; BD Biosciences) to block FcRs for 15 min at room temperature (RT) and then stained with tetramer (5 µg/mL) in FACS buffer (0.05% sodium azide and 1% FBS in PBS) for 30 min at 4°C (BDC2.5mi/IA^g7^ tetramer) or 1 h at 37 °C (for all insulin epitope-based pMHCII tetramers), washed, and incubated with the corresponding antibodies. If only tetramer+ quantification was required, staining was performed with anti-CD4-FITC (GK1.5, eBioscience) and anti-PerCP.Cy5.5-conjugated anti-B220 (RA3-6B2, BD Bioscience). To determine antigen-specific cell phenotype, additional antibodies were used: anti-PD1-BV421 (J43, BD Bioscience), anti-ICOS-APC (C398.4A, ThermoFisher), anti-LAG-3-APC (C9B7W, BioLegend). LAG-3 was stained with APC-conjugated Ab, followed by biotin-conjugated anti-APC and streptavidin-APC. Cells were washed, fixed in 1% paraformaldehyde (PFA) in PBS and analyzed with FACSCanto or LSR Fortessa flow cytometers. Analysis was done using FlowJo software (FlowJo, BD Biosciences, San Diego, CA, USA). TCR-transduced JurMA cell lines were stained in 50 μl FACS buffer (2% FBS in PBS) at 37°C for 1 hour with 10 μg/ml (conventional pMHCII-tetramer-PE) or 20 μg/ml (KIH-based pMHCII-tetramer-PE). Samples were acquired in 200 μl FACS buffer in LSR Fortessa.

### Nanoparticle synthesis

Maleimide-functionalized, pegylated iron oxide NPs (PFM series) were produced in a single-step thermal decomposition in the absence of surfactants as described (1). Briefly, 3 g Maleimide-PEG (2 kDa MW, Jenkem Tech USA) were melted in a 50 mL round bottom flask at 100°C and then mixed with 7 mL of benzyl ether and 2 mmol Fe(acac)_3_. The reaction was stirred for 1 h and heated to 260°C with reflux for 2 h. The mixture was cooled to room temperature and mixed with 30 mL water. Insoluble materials were removed by centrifugation at 2,000xg for 30 min. The NPs were purified using magnetic (MACS) columns (Miltenyi Biotec, Auburn, CA, USA) and stored in water at room temperature or 4°C. The concentration of iron was determined spectrophotometrically at 410 nm in 2N hydrochloric acid (HCl).

### pMHCII conjugation to NPs

pMHC conjugation to maleimide-functionalized NPs (PFM) was done *via* the free C-terminal Cys engineered into the MHCα chain/knob. Briefly, pMHCs were mixed with NPs in 40 mM phosphate buffer, pH 6.0, containing 2 mM ethylenediaminetetraacetic acid (EDTA), 150 mM NaCl, and incubated overnight at room temperature. pMHC-conjugated NPs were purified by magnetic separation and concentrated by ultrafiltration through Amicon Ultra-15 (100 kDa cut-off) (Merck KGaA, Darmstadt, Germany) and stored in PBS.

### NP characterization

The size and dispersity of unconjugated and pMHC-conjugated NPs were assessed *via* transmission electron microscopy (TEM, Hitachi H7650, Hitachi, Chiyoda, Tokio, Japan) and dynamic light scattering (DLS, Zetasizer, Malvern Panalytical, Spectris, Egham, UK). Pegylated and pMHC-NPs were analyzed *via* 0.8% agarose gel electrophoresis, native- and denaturing 10% SDS-PAGE. To quantify pMHC valency, we measured the pMHC concentration of the pMHC-NP preps using the Bradford assay (Thermo Scientific, Waltham, MA, USA).

### pMHCII-NP therapy of NOD mice

Cohorts of 10 week-old pre-diabetic female NOD mice were injected i.v. with pMHCII-coated NPs in PBS twice a week for 5 weeks. Treatment-induced formation and expansion of cognate TR1-like cells were assessed by flow cytometry, using the markers described above (2). Experiments in diabetic mice involved following cohorts of 10 week-old female NOD/Ltj for diabetes development by measuring blood glucose levels with Accucheck Strips (La Roche, Basel, Switzerland) twice a wk. Mice displaying glucose measurements >11mM were considered diabetic and treated twice weekly with 20 µg pMHCII-NPs or vehicle (PBS) for 10 weeks, until stably normoglycemic or until hyperglycemia was considered irreversible (3 measurements >25mM).

### SMARTseq2 scRNAseq

One-cell sorting of tetramer+ and tetramer– cells from splenic CD4+ T cell pools of pMHCII-NP-treated NOD mice was done in SMARTseq2 96-well plates containing lysis buffer. Specifically, we sorted 768 tetramer– cells from splenic CD4+ T cells pooled from 5 mice treated with Ins_12-20_-R1/IA^g7^-NPs, 768 tetramer+ cells from mice treated with each of the three InsB epitope-based pMHCII-NPs (n=5-6 mice per pMHCII-NP type), and 1,152 tetramer+ cells from BDC2.5mi/IA^g7^-NP-treated mice (n=5 mice). Plates were spun and frozen after sorting. Full-length single-cell RNA sequencing libraries were prepared using a modified Smart-Seq protocol (27). Reverse transcription was done using SuperScrpit II (Invitrogen) in the presence of oligo-dT30VN, template-switching oligonucleotides and betaine. The cDNA was amplified using the KAPA Hifi Hotstart ReadyMix (Kapa Biosystems, La Roche, Basel, Switzerland), ISPCR primer and 25 cycles of amplification. Following purification with Agencourt AMPure XP beads (Beckman Coulter, Brea, CA, USA), product size distribution and quantity were assessed on a Bioanalyzer using a High Sensitivity DNA Kit (Agilent Technologies, Santa Clara, CA, USA). 200 pg of the amplified cDNA was fragmented and amplified with indexed Nextera^®^ PCR primers. Products were purified twice with Agencourt AMPure XP beads and quantified again using a Bioanalyzer High Sensitivity DNA Kit. Sequencing of Nextera^®^ libraries from 384 cells was carried out using one sequencing lane on an Illumina HiSeq2500 v4 or HiSeq4000 to 500K reads/cell.

### Bioinformatics

Quality check of the reads was performed with the FastQC v0.11.8 software (Babraham Bioinformatics, Babraham, Cambridge, UK). Only successfully sequenced cells expressing more than 300 genes and <10% mitochondrial content were analyzed (n=181 for tetramer– cells; n=97 for InsB_12-20_-CT-R1/IA^g7^ tetramer+ cells; n=93 for InsB_13-21_-R2/IA^g7^ tetramer+ cells; n=156 for InsB_10-23_-CT-R3/IA^g7^ tetramer+ cells; and n=190 for BDC2.5mi/IAg7 tetramer+ cells). Reads were aligned with STAR v2.5.4b (28) on the mouse genome reference GRCm38 with Gencode M21 annotations. Gene expression was estimated with RSEM v1.3.0 (29). Downstream analysis including dimensionality reduction (tSNE), cluster analysis (K-means) and differential expression analysis, was performed in Seurat R package (30). TCR sequences were reconstructed to infer clonality with TraCeR v.0.5.1 (12). We used TraCeR for mapping the reads to mouse Gencode release M9 (GRCm38). Parameters *–loci A B* were used to allow reconstruction of TCRα and β regions in each cell. Other parameters chosen were *–kmerLength 31* and *–max_junc_len 50*. For clonotype classification, TCRα and TCRβ sequences recovered from single cells were, separately, aligned against a recombinome created from IMGT (The International ImMunoGeneTics information system, http://www.imgt.org). This recombinome file contains each possible V(D)J combination for each TCR chain separated by N nucleotides in order to align reads that spanned diverse junctional sequences. Each TCR sequence is then identified by its V element, the junctional nucleotide sequence and the J gene name. All cells containing the same identifier (V-junction-J) are considered the same clonotype.

### TCR signaling in TCRαβ/mCD4-transfected JurMA cells

Retroviruses encoding mouse CD4 (mCD4) or a P2A sequence tethering TCRα and TCRβ open-reading frames upstream of an IRES-eGFP cassette were synthesized by Vector Builder (Chicago, IL, USA). The human CD3+/TCRβ– JurMA (Jurkat) reporter cell line (expressing NFAT-driven luciferase) was sequentially transduced with retroviruses encoding mCD4 and the different TCRαβ. eGFP and mCD4 double-positive cells were sorted by flow cytometry. To measure NFAT-driven expression of luciferase, 100,000 cells were plated on pMHCII-coated (20 µg/ml) 96-well plates in 200 µl of DMEM (Sigma-Aldrich, St. Louis, Missouri, USA) supplemented with 10% FBS (Sigma-Aldrich) for 24h. Triplicates were pooled and lysed in 90 µl Cell Culture Lysis Reagent (Promega, Madison, WI, USA) for 20 minutes and 30 µl of cell lysate was incubated with 100 µl of Luciferase Assay Reagent (Promega) in opaque white plates (Greiner Bio One International GmbH, Kremsmünster, Austria) using a Veritas™ Microplate Luminometer (Promega) with injectors. Luciferase activity was expressed as relative luminescence units (RLUs), normalized to the luciferase activity of non-stimulated cells, and reported as stimulation indexes relative to negative control.

### Statistics

Statistical analyses of single cell transcriptional data were done in Seurat. Differences in gene expression by negative binomial test were considered statistically significant when |FC|≥4 and FDR ≤ 0.01. Statistical correlations in gene expression differences between groups were tested *via* Pearson’s correlation. Overrepresentation of significantly expressed TFH- and TR1-relevant genes in different clusters were assessed *via* Chi-square. Differences in blood glucose levels among different cohorts were analyzed *via* two-way ANOVA.

## Data availability statement

The datasets presented in this study can be found in online repositories. The names of the repository and accession number(s) can be found below: GSE173601 and GSE173824 (GEO). https://www.ncbi.nlm.nih.gov/geo/query/acc.cgi?acc=GSE173601, https://www.ncbi.nlm.nih.gov/geo/query/acc.cgi?acc=GSE173824.

## Ethics statement

The animal experiments described herein were approved by the University of Calgary and Universitat de Barcelona Animal Care Committees.

## Author contributions

PSo generated the data for **Figures 2**–**5**, **Tables 1**–**3** and **Supplementary Figures 1, 2**. JY generated the data for **Figure 7**. DP generated the data for **Figures 1**, **6**. PSo, JG, and JoM carried out the bioinformatic analyses of the data. PSo, DP, NG, JaM, DM, CF, and YY produced NPs, pMHCIIs, pMHCII-NP and pMHCII tetramers. PSe engineered the constructs used herein and co-supervised IDIBAPS co-authors with PSa. PSa designed the study, supervised its execution and wrote the manuscript with PSo. All authors contributed to the article and approved the submitted version.
