# Supplementary material for: Transcriptional re-programming of insulin B-chain epitope-specific T-follicular helper cells into anti-diabetogenic T-regulatory type-1 cells
Source: Front Immunol. 2023 Apr 19;14:1177722. doi: 10.3389/fimmu.2023.1177722 (PMC10154693; doi:10.3389/fimmu.2023.1177722)
Supplement: Supplementary file 1 [file Presentation_1.pdf]

Suppl. Fig. 1

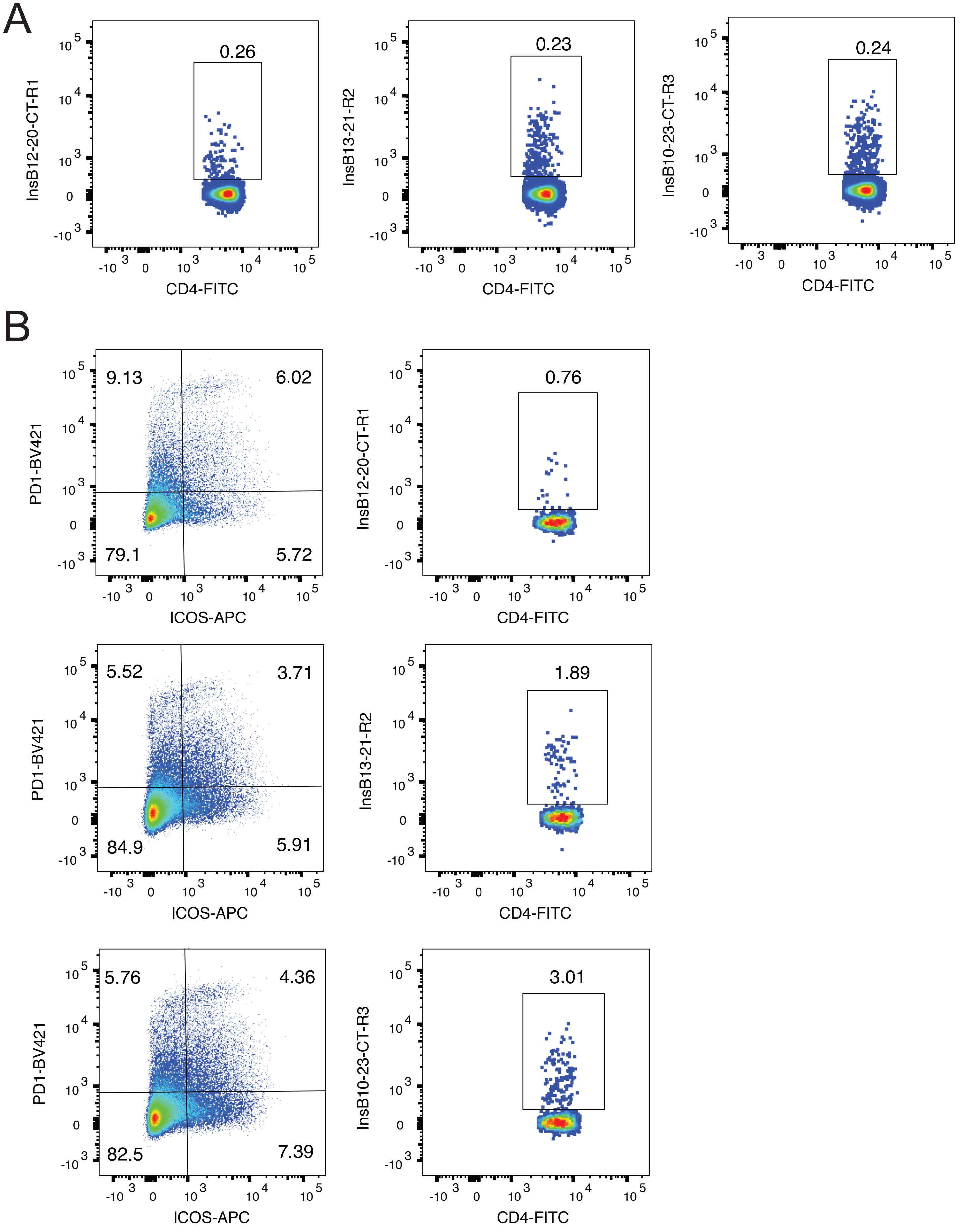

A
InsB12-20-CT-R1
0.26
CD4-FITC
InsB13-21-R2
0.23
CD4-FITC
InsB10-23-CT-R3
0.24
CD4-FITC
B
PD1-BV421
9.13
6.02
79.1
5.72
ICOS-APC
InsB12-20-CT-R1
0.76
CD4-FITC
PD1-BV421
5.52
3.71
84.9
5.91
ICOS-APC
InsB13-21-R2
1.89
CD4-FITC
PD1-BV421
5.76
4.36
82.5
7.39
ICOS-APC
InsB10-23-CT-R3
3.01
CD4-FITC

Supplementary Figure 1. A, pMHCII tetramer/CD4-FITC staining profiles for pooled splenocytes from pMHCII-NP-treated mice (n=5 mice/group). Profiles correspond to CD4+B220– cells. **B,** Gating strategy for sorting Tetramer+ TR1-like CD4+ T-cells for scRNAseq studies. Left, ICOS (APC) and PD-1 (BV421) staining within the splenic CD4+B220- T-cell pool. Right, pMHCII tetramer staining within the ICOS+/PD-1+ gate (Q2 in middle plot). Tet+/ICOS+/PD-1+ cells were sorted for transcriptomic studies.

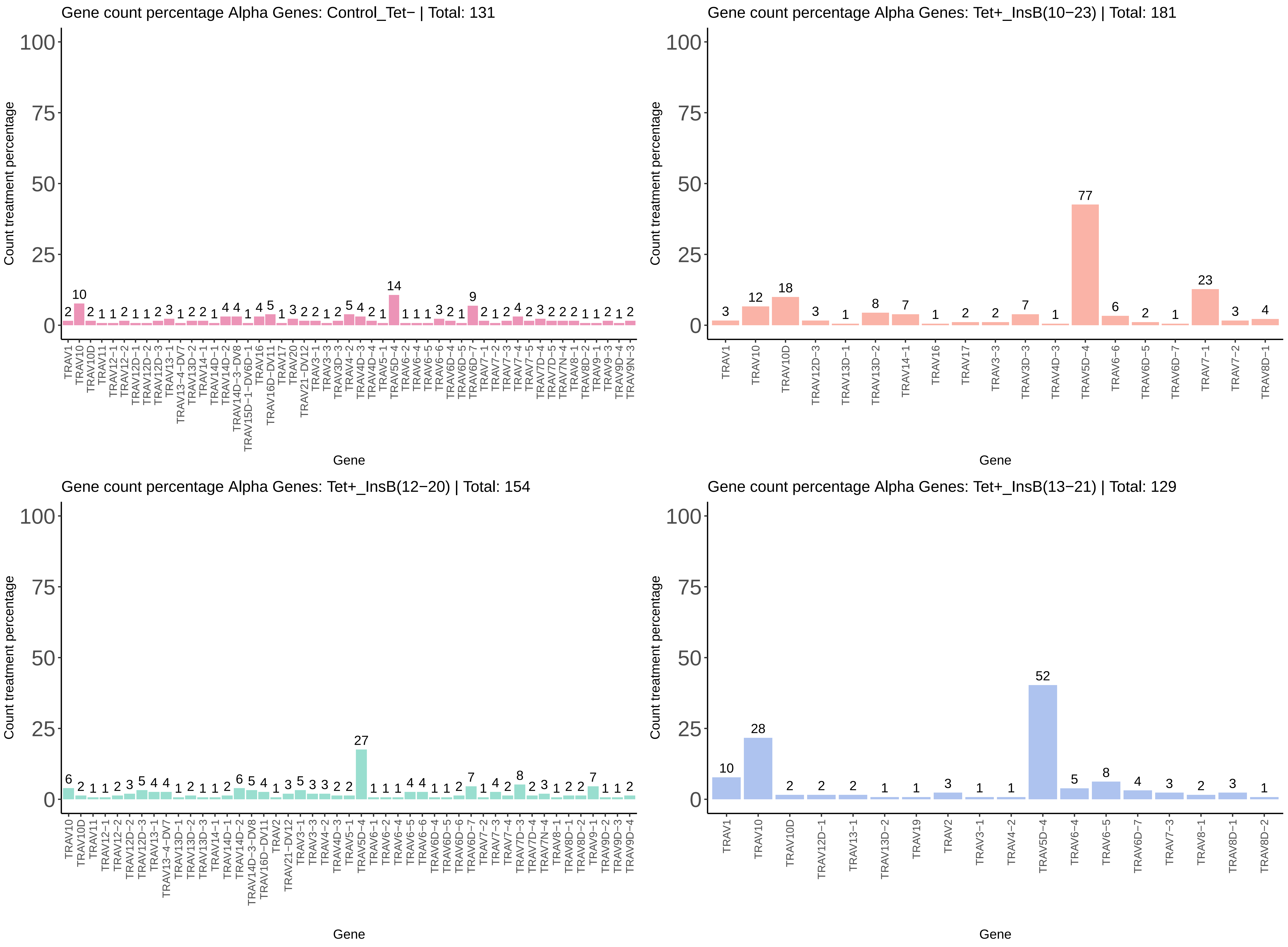

Gene count percentage Alpha Genes: Control_Tet− | Total: 131
Count treatment percentage
0
25
50
75
100
2 10 2 1 1 2 1 1 2 3 1 2 2 1 4 4 1 4 5 1 3 2 2 1 2 5 4 2 1 14 1 1 1 3 2 1 9 2 1 2 4 2 3 2 2 2 1 1 2 1 2
TRAV1 TRAV10 TRAV10D TRAV11 TRAV12−1 TRAV12−2 TRAV12D−1 TRAV12D−2 TRAV12D−3 TRAV13−1 TRAV13−4−DV7 TRAV13D−2 TRAV14−1 TRAV14D−1 TRAV14D−2 TRAV14D−3−DV8 TRAV15D−1−DV6D−1 TRAV16 TRAV16D−DV11 TRAV17 TRAV20 TRAV21−DV12 TRAV3−1 TRAV3−3 TRAV3D−3 TRAV4−2 TRAV4D−3 TRAV4D−4 TRAV5−1 TRAV5D−4 TRAV6−2 TRAV6−4 TRAV6−5 TRAV6−6 TRAV6D−4 TRAV6D−5 TRAV6D−7 TRAV7−1 TRAV7−2 TRAV7−3 TRAV7−4 TRAV7−5 TRAV7D−4 TRAV7D−5 TRAV7N−4 TRAV8−1 TRAV8D−2 TRAV9−1 TRAV9−3 TRAV9D−4 TRAV9N−3
Gene
Gene count percentage Alpha Genes: Tet+_InsB(10−23) | Total: 181
Count treatment percentage
0
25
50
75
100
3 12 18 3 1 8 7 1 2 2 7 1 77 6 2 1 23 3 4
TRAV1 TRAV10 TRAV10D TRAV12D−3 TRAV13D−1 TRAV13D−2 TRAV14−1 TRAV16 TRAV17 TRAV3−3 TRAV3D−3 TRAV4D−3 TRAV5D−4 TRAV6−6 TRAV6D−5 TRAV6D−7 TRAV7−1 TRAV7−2 TRAV8D−1
Gene
Gene count percentage Alpha Genes: Tet+_InsB(12−20) | Total: 154
Count treatment percentage
0
25
50
75
100
6 2 1 1 2 3 5 4 4 1 2 1 1 2 6 5 4 1 3 5 3 3 2 2 27 1 1 1 4 4 1 1 2 7 1 4 2 8 2 3 1 2 2 7 1 1 2
TRAV10 TRAV10D TRAV11 TRAV12−1 TRAV12−2 TRAV12D−2 TRAV12D−3 TRAV13−1 TRAV13−4−DV7 TRAV13D−1 TRAV13D−2 TRAV13D−3 TRAV14−1 TRAV14D−1 TRAV14D−2 TRAV14D−3−DV8 TRAV16D−DV11 TRAV2 TRAV21−DV12 TRAV3−1 TRAV3−3 TRAV4−2 TRAV4D−3 TRAV5−1 TRAV5D−4 TRAV6−1 TRAV6−2 TRAV6−4 TRAV6−5 TRAV6−6 TRAV6D−4 TRAV6D−5 TRAV6D−6 TRAV6D−7 TRAV7−2 TRAV7−3 TRAV7−4 TRAV7D−3 TRAV7D−4 TRAV7N−4 TRAV8−1 TRAV8D−1 TRAV8D−2 TRAV9−1 TRAV9D−2 TRAV9D−3 TRAV9D−4
Gene
Gene count percentage Alpha Genes: Tet+_InsB(13−21) | Total: 129
Count treatment percentage
0
25
50
75
100
10 28 2 2 2 1 1 3 1 1 52 5 8 4 3 2 3 1
TRAV1 TRAV10 TRAV10D TRAV12D−1 TRAV13−1 TRAV13D−2 TRAV19 TRAV2 TRAV3−1 TRAV4−2 TRAV5D−4 TRAV6−4 TRAV6−5 TRAV6D−7 TRAV7−3 TRAV8−1 TRAV8D−1 TRAV8D−2
Gene

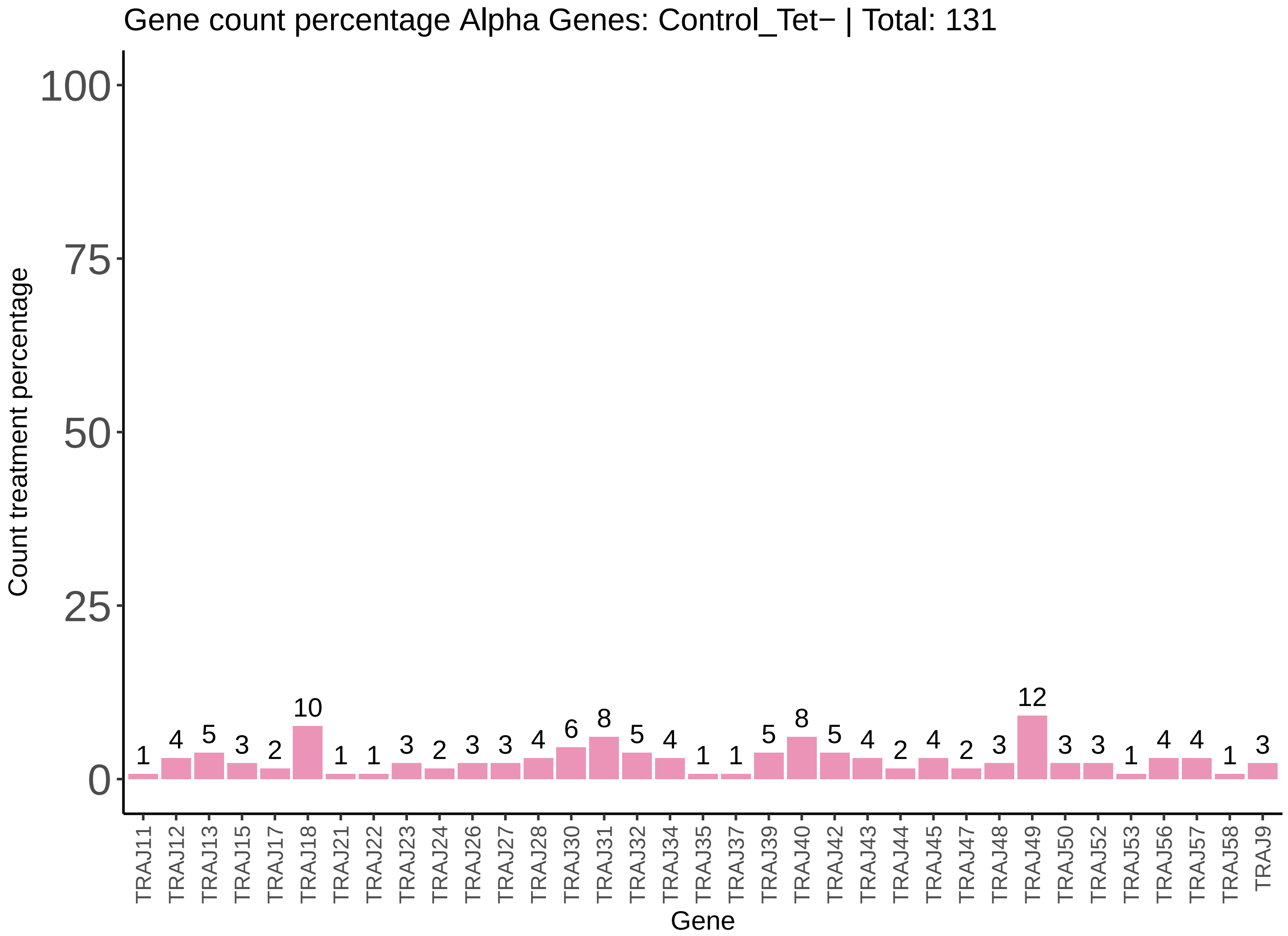
Gene count percentage Alpha Genes: Control_Tet− | Total: 131
Count treatment percentage
Gene
TRAJ11 TRAJ12 TRAJ13 TRAJ15 TRAJ17 TRAJ18 TRAJ21 TRAJ22 TRAJ23 TRAJ24 TRAJ26 TRAJ27 TRAJ28 TRAJ30 TRAJ31 TRAJ32 TRAJ34 TRAJ35 TRAJ37 TRAJ39 TRAJ40 TRAJ42 TRAJ43 TRAJ44 TRAJ45 TRAJ47 TRAJ48 TRAJ49 TRAJ50 TRAJ52 TRAJ53 TRAJ56 TRAJ57 TRAJ58 TRAJ9

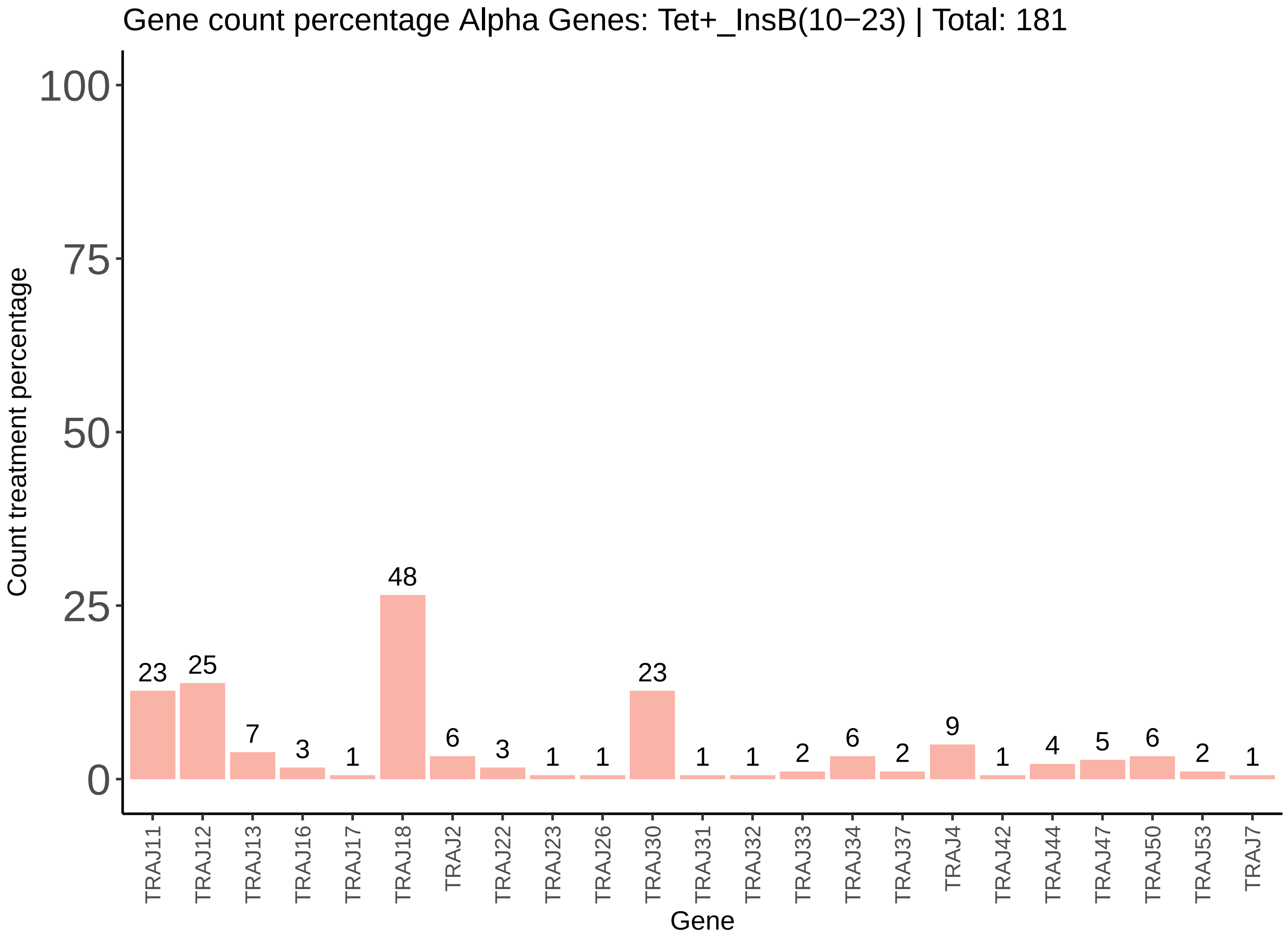
Gene count percentage Alpha Genes: Tet+_InsB(10−23) | Total: 181
Count treatment percentage
Gene
TRAJ11 TRAJ12 TRAJ13 TRAJ16 TRAJ17 TRAJ18 TRAJ2 TRAJ22 TRAJ23 TRAJ26 TRAJ30 TRAJ31 TRAJ32 TRAJ33 TRAJ34 TRAJ37 TRAJ4 TRAJ42 TRAJ44 TRAJ47 TRAJ50 TRAJ53 TRAJ7

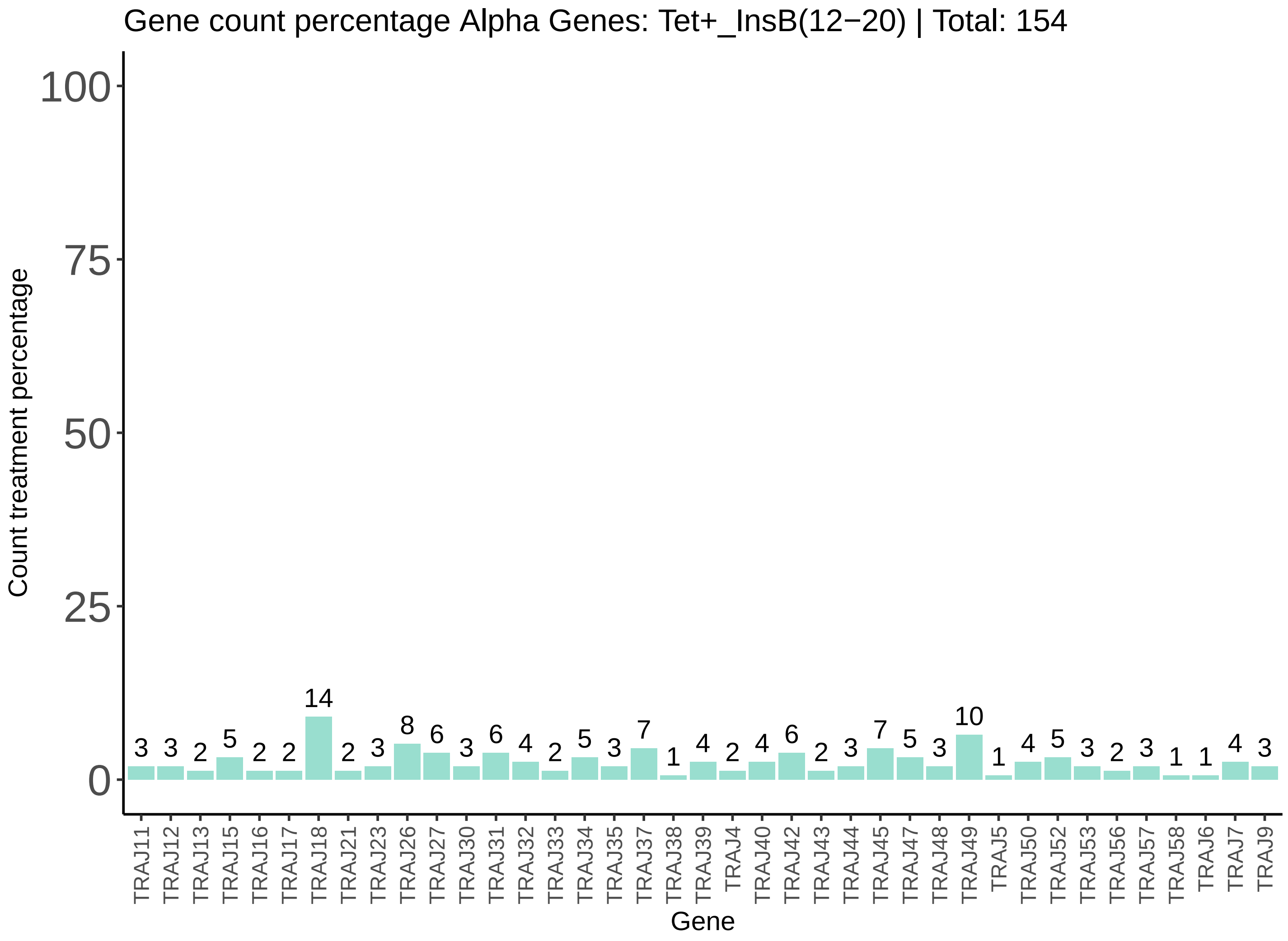
Gene count percentage Alpha Genes: Tet+_InsB(12−20) | Total: 154
Count treatment percentage
Gene
TRAJ11 TRAJ12 TRAJ13 TRAJ15 TRAJ16 TRAJ17 TRAJ18 TRAJ21 TRAJ23 TRAJ26 TRAJ27 TRAJ30 TRAJ31 TRAJ32 TRAJ33 TRAJ34 TRAJ35 TRAJ37 TRAJ38 TRAJ39 TRAJ4 TRAJ40 TRAJ42 TRAJ43 TRAJ44 TRAJ45 TRAJ47 TRAJ48 TRAJ49 TRAJ5 TRAJ50 TRAJ52 TRAJ53 TRAJ56 TRAJ57 TRAJ58 TRAJ6 TRAJ7 TRAJ9

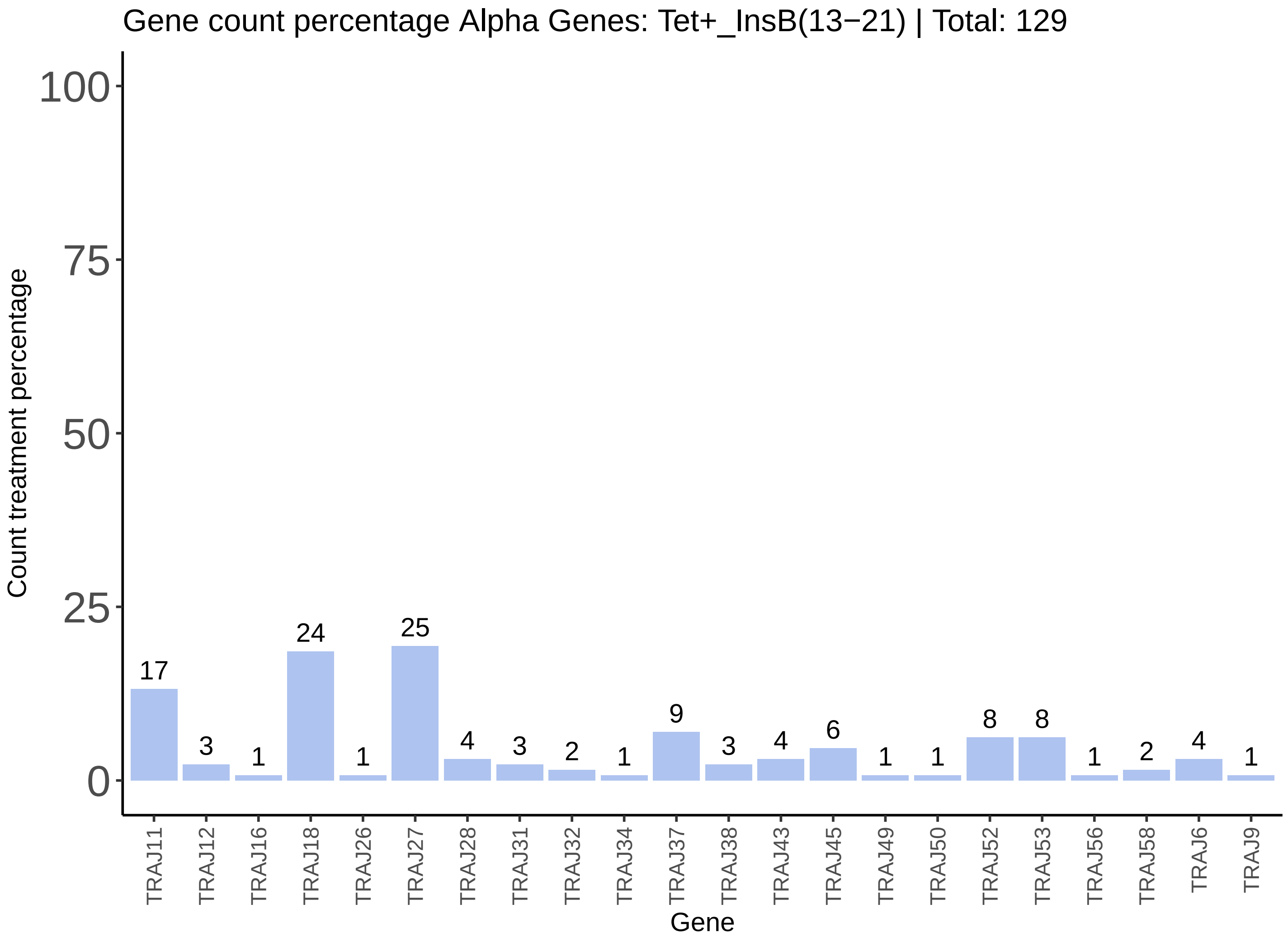
Gene count percentage Alpha Genes: Tet+_InsB(13−21) | Total: 129
Count treatment percentage
Gene
TRAJ11 TRAJ12 TRAJ16 TRAJ18 TRAJ26 TRAJ27 TRAJ28 TRAJ31 TRAJ32 TRAJ34 TRAJ37 TRAJ38 TRAJ43 TRAJ45 TRAJ49 TRAJ50 TRAJ52 TRAJ53 TRAJ56 TRAJ58 TRAJ6 TRAJ9

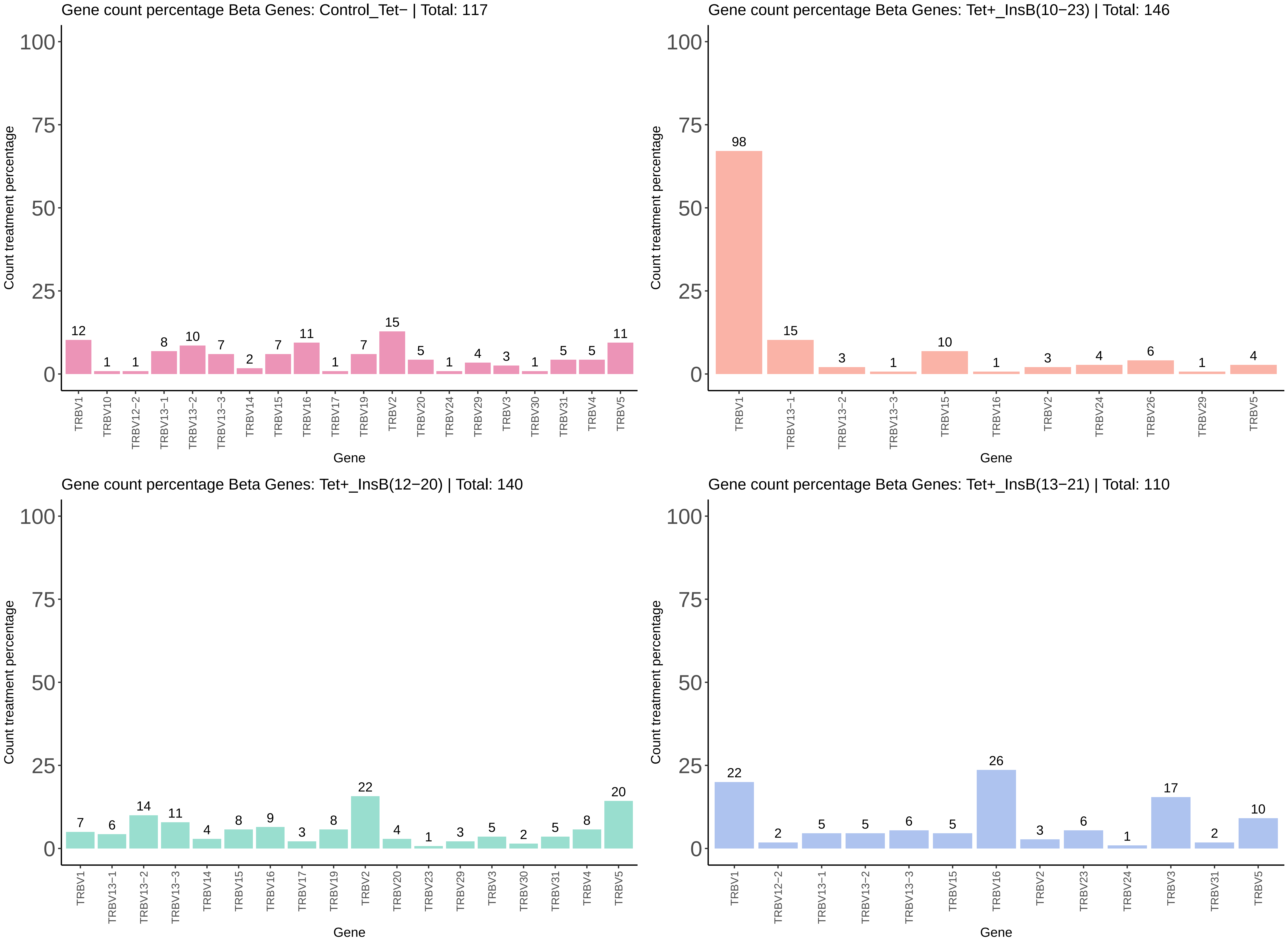
Gene count percentage Beta Genes: Control_Tet− | Total: 117
Count treatment percentage
Gene
0
25
50
75
100
TRBV1 12
TRBV10 1
TRBV12−2 1
TRBV13−1 8
TRBV13−2 10
TRBV13−3 7
TRBV14 2
TRBV15 7
TRBV16 11
TRBV17 1
TRBV19 7
TRBV2 15
TRBV20 5
TRBV24 1
TRBV29 4
TRBV3 3
TRBV30 1
TRBV31 5
TRBV4 5
TRBV5 11
Gene count percentage Beta Genes: Tet+_InsB(10−23) | Total: 146
Count treatment percentage
Gene
TRBV1 98
TRBV13−1 15
TRBV13−2 3
TRBV13−3 1
TRBV15 10
TRBV16 1
TRBV2 3
TRBV24 4
TRBV26 6
TRBV29 1
TRBV5 4
Gene count percentage Beta Genes: Tet+_InsB(12−20) | Total: 140
Count treatment percentage
Gene
TRBV1 7
TRBV13−1 6
TRBV13−2 14
TRBV13−3 11
TRBV14 4
TRBV15 8
TRBV16 9
TRBV17 3
TRBV19 8
TRBV2 22
TRBV20 4
TRBV23 1
TRBV29 3
TRBV3 5
TRBV30 2
TRBV31 5
TRBV4 8
TRBV5 20
Gene count percentage Beta Genes: Tet+_InsB(13−21) | Total: 110
Count treatment percentage
Gene
TRBV1 22
TRBV12−2 2
TRBV13−1 5
TRBV13−2 5
TRBV13−3 6
TRBV15 5
TRBV16 26
TRBV2 3
TRBV23 6
TRBV24 1
TRBV3 17
TRBV31 2
TRBV5 10

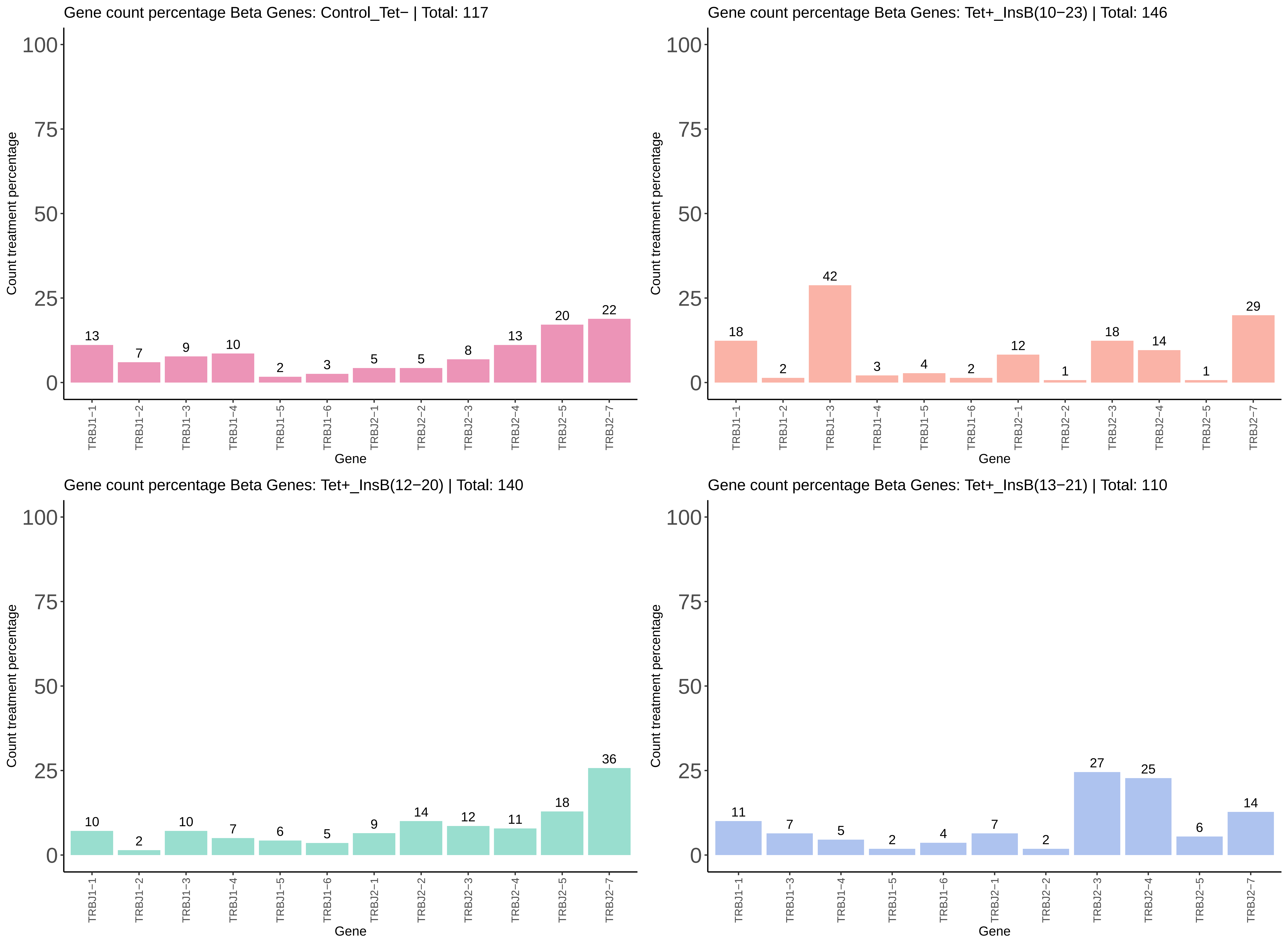

Gene count percentage Beta Genes: Control_Tet− | Total: 117
Count treatment percentage
100
75
50
25
0
13
7
9
10
2
3
5
5
8
13
20
22
TRBJ1−1
TRBJ1−2
TRBJ1−3
TRBJ1−4
TRBJ1−5
TRBJ1−6
TRBJ2−1
TRBJ2−2
TRBJ2−3
TRBJ2−4
TRBJ2−5
TRBJ2−7
Gene
Gene count percentage Beta Genes: Tet+_InsB(10−23) | Total: 146
Count treatment percentage
100
75
50
25
0
18
2
42
3
4
2
12
1
18
14
1
29
TRBJ1−1
TRBJ1−2
TRBJ1−3
TRBJ1−4
TRBJ1−5
TRBJ1−6
TRBJ2−1
TRBJ2−2
TRBJ2−3
TRBJ2−4
TRBJ2−5
TRBJ2−7
Gene
Gene count percentage Beta Genes: Tet+_InsB(12−20) | Total: 140
Count treatment percentage
100
75
50
25
0
10
2
10
7
6
5
9
14
12
11
18
36
TRBJ1−1
TRBJ1−2
TRBJ1−3
TRBJ1−4
TRBJ1−5
TRBJ1−6
TRBJ2−1
TRBJ2−2
TRBJ2−3
TRBJ2−4
TRBJ2−5
TRBJ2−7
Gene
Gene count percentage Beta Genes: Tet+_InsB(13−21) | Total: 110
Count treatment percentage
100
75
50
25
0
11
7
5
2
4
7
2
27
25
6
14
TRBJ1−1
TRBJ1−3
TRBJ1−4
TRBJ1−5
TRBJ1−6
TRBJ2−1
TRBJ2−2
TRBJ2−3
TRBJ2−4
TRBJ2−5
TRBJ2−7
Gene

Supplementary Figure 2. TRAV, TRAJ, TRBV and TRBJ usage. Histogram plots comparing the number of TCRα and TCRβ sequences carrying different Vα, Jα, Vβ and Jβ-coding elements.

Suppl. Fig. 3

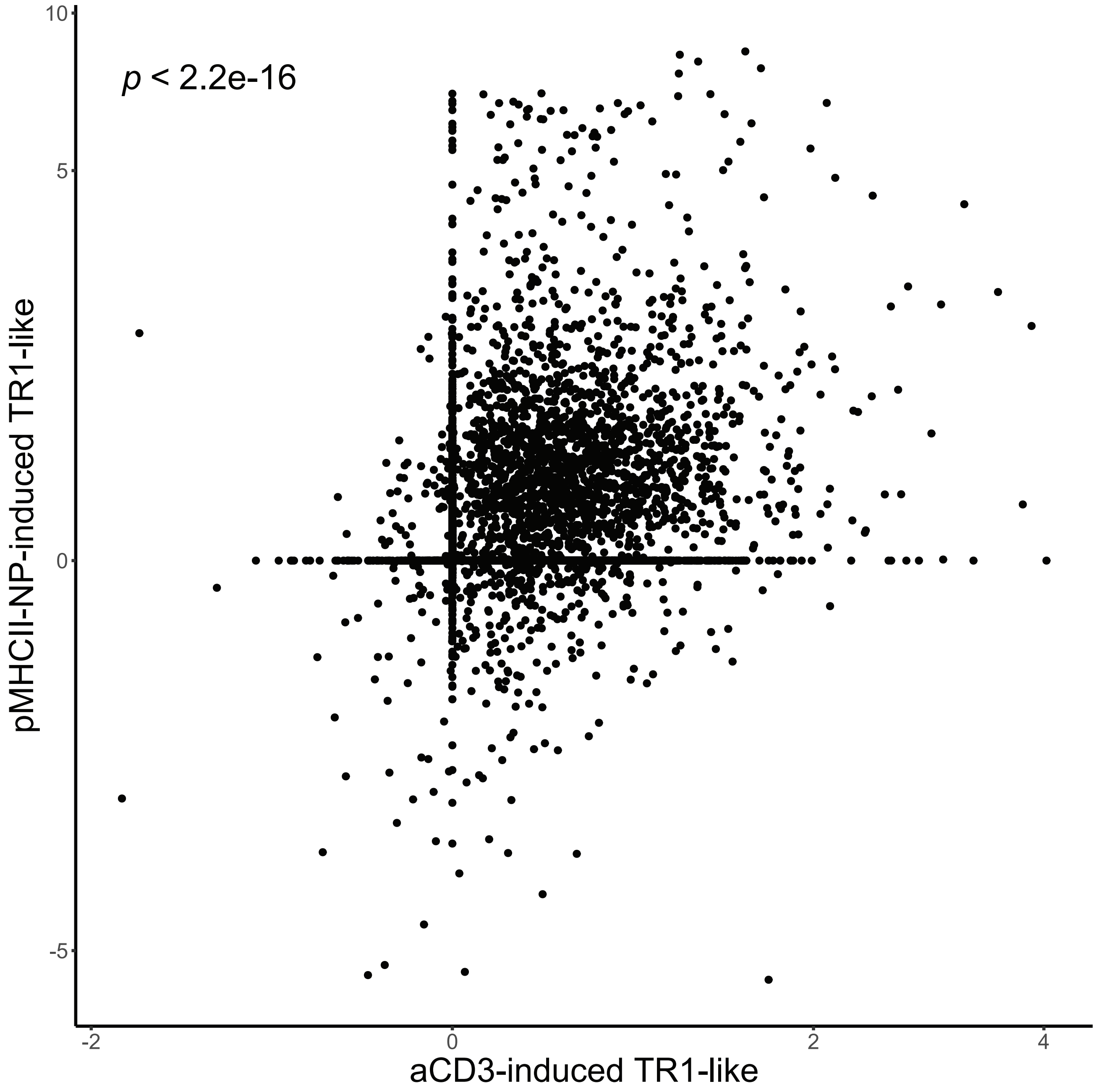

p < 2.2e-16
pMHCII-NP-induced TR1-like
aCD3-induced TR1-like
10
5
0
-5
-2
0
2
4

Supplementary Figure 3. pMHCII-NP-induced TR1-like cells are transcriptionally similar to anti-CD3 mAb-induced TR1-like cells. Correlation between the levels of gene expression between TR1-like (cluster #2) cells from mice treated with $InsB_{12-20}$-CT-R1/IA^{g7}-NP, $InsB_{13-21}$-R2/IA^{g7}-NP, $InsB_{10-23}$-CT-R3/IA^{g7}-NP and BDC2.5mi/IA^{g7}-NP (cluster #2) and the TR1-like cells induced by anti-CD3 mAb (cluster #2 in ref. 5) (relative to Tconv cells). The P value refers to the Pearson correlation coefficient.
